# Effects of Replacing Alfalfa with *Sesbania cannabina* Hay on Growth Performance, Rumen Fermentation, and Rumen Microecology in Fattening Lambs

**DOI:** 10.3390/ani16172745

**Published:** 2026-09-02

**Authors:** Yin Ma, Zesheng Yan, Chenglong Yan, Shihong Yang, Xian Deng, Ding Tang, Xiaofeng Cao, Qiyu Diao, Guishan Xu

**Affiliations:** 1 College of Animal Science and Technology, Tarim University, Alar 843300, China; 18189617850@163.com (Y.M.);; 2 Institute of Genetics and Developmental Biology, Chinese Academy of Sciences, Beijing 100101, China; 3 Feed Research Institute, Chinese Academy of Agricultural Sciences, Beijing 100081, China; 4 Key Laboratory of Livestock and Forage Resources Utilization in the Tarim Basin, Ministry of Agriculture and Rural Affairs, Alar 843300, China

**Keywords:** *Sesbania cannabina* hay, alfalfa replacement, fattening lambs, nutrient digestibility, ruminal fermentation, multi-omics

## Abstract

This study evaluated the effects of replacing alfalfa (*Medicago sativa*) with *Sesbania cannabina* hay on growth performance, nutrient digestibility, and ruminal microecology in fattening lambs. Forty 3-month-old Aohu crossbred lambs (initial BW: 22.08 ± 0.31 kg) received five isoenergetic, isonitrogenous diets in which *S. cannabina* hay replaced 0% (CON), 25%, 50%, 75%, or 100% of alfalfa during a 60-day trial. Growth traits, nutrient digestibility, rumen fermentation, enzyme activities, serum biochemical parameters, ruminal microbiota, and metabolomic profiles were evaluated. Substitution did not affect final body weight, average daily gain, or feed conversion ratio (*p* > 0.05), but nutrient utilization and ruminal fermentation differed among replacement treatments. The 50–100% replacement groups showed higher digestibility of crude protein, ether extract, and fiber, together with higher ruminal protease, cellulase, hemicellulase, and α-amylase activities (*p* < 0.05). Total volatile fatty acid concentrations were higher in the 50–100% replacement groups, whereas the acetate-to-propionate ratio was lower in all substituted groups. Serum biochemical parameters indicated changes related to protein metabolism without evidence of adverse health effects. Multi-omics analyses revealed distinct differences in ruminal bacterial communities and metabolic profiles among replacement treatments. The 25% replacement group showed relatively limited microbial and metabolic changes. The 75% replacement group exhibited greater bacterial diversity, enrichment of *Prevotellaceae_NK3B31_group* and *Streptococcus*, coordinated microbe–metabolite associations, and changes in bile acid and cholesterol metabolism. Complete substitution was associated with more extensive microbial remodeling and lower abundances of several functional metabolites, without additional benefits to growth performance.

## Simple Summary

Alfalfa is widely used in lamb diets, but alternative forage resources are needed. This study tested whether *Sesbania cannabina* hay could replace alfalfa in diets for fattening lambs. Replacing alfalfa with *S. cannabina* hay did not adversely affect growth performance or feed efficiency while improving nutrient digestibility, rumen enzyme activities, and fermentation and altering the rumen microbial community and metabolic profile. Among the tested diets, the 75% replacement was associated with coordinated changes in rumen fermentation and microbial–metabolite responses. These findings suggest that *S. cannabina* hay can partially replace alfalfa in diets for fattening lambs without compromising growth performance while influencing nutrient utilization, rumen fermentation, and rumen microecology.

## 1. Introduction

Global agricultural systems are increasingly challenged by soil salinization, freshwater scarcity, and climatic instability, which restrict the productivity and nutritional quality of conventional forage crops worldwide [1]. At the same time, the expansion of intensive livestock production has increased the demand for high-quality roughage, while competition for arable land and water resources has constrained the supply of traditional forage resources [2]. Under these conditions, identifying salt-tolerant, high-yielding, and nutritionally suitable alternative forages is increasingly important for improving the resilience of ruminant production systems and promoting the productive use of marginal saline–alkali land.

Alfalfa (*Medicago sativa*) is a widely cultivated perennial leguminous forage because of its high crude protein content, favorable digestibility, and palatability and is commonly referred to as the “Queen of Forages” [3]. However, alfalfa production can be substantially constrained in arid and semi-arid regions by soil salinization, freshwater scarcity, and competition for high-quality agricultural land [4]. These limitations have stimulated interest in alternative forage species capable of maintaining acceptable biomass yield and nutritional value under adverse environmental conditions [5].

*Sesbania cannabina* (*Retz.*) *Poir.* is a salt- and drought-tolerant leguminous forage species that can grow on marginal saline land [6]. It exhibits relatively high biomass productivity, and crude protein concentrations of approximately 22–24% have been reported under certain growth and harvest conditions [7], indicating its potential as an alternative forage resource. However, its nutrient composition can vary considerably with growth stage, cultivar, environmental conditions, and post-harvest processing. In addition, *S. cannabina* has biological nitrogen-fixation capacity and may contribute to improvements in soil structure, reductions in surface salt accumulation, and increases in soil organic matter and available nitrogen [8]. However, crude protein concentration alone does not determine feeding value. Fiber composition, digestibility, mineral profile, palatability, and secondary metabolites may also influence rumen fermentation, voluntary feed intake, and animal performance. Similar to other unconventional forages, *S. cannabina* may show variation in nutrient composition among growth stages and environmental conditions and contains tannins, phenolic compounds, and other secondary metabolites that may limit its use at high dietary inclusion levels [9].

Compared with other emerging alternative forage resources, the crude protein concentration of *S. cannabina* is comparable to that reported for *Azolla pinnata* as a sustainable feed resource for sheep and is higher than that of many halophytic roughages with relatively low crude protein concentrations [10]. Recent evidence also indicates that unconventional protein sources such as microalgae can produce variable effects on nutrient digestibility, rumen fermentation, and voluntary intake depending on species, processing methods, and dietary inclusion levels [11]. These comparisons suggest that the feeding value of *S. cannabina* should be evaluated not only on the basis of crude protein concentration but also in relation to fiber characteristics, secondary metabolites, palatability, and appropriate substitution level.

Fresh *S. cannabina* forage can be offered directly to animals or processed into silage or hay. Farghaly et al. [12] reported that feeding fresh *S. cannabina* improved meat quality and tenderness in lambs, with acceptable color characteristics of meat and adipose tissue. Gebreyowhans and Zegeye [13] demonstrated that dietary inclusion of *S. cannabina* hay exerted significant effects on crude protein digestibility (CPD), neutral detergent fiber digestibility (NDFD) and acid detergent fiber digestibility (ADFD), and simultaneously increased total DM intake (DMI) and milk yield. In a sheep feeding trial conducted by Khandaker et al. [14], supplementation with 10% and 20% *S. cannabina* in wheat straw-based diets elevated DM intake of wheat straw. Ruminal fluid pH decreased linearly with increasing *S. cannabina* inclusion, and the optimum utilization efficiency of wheat straw by sheep was achieved at the 20% inclusion level. Makau et al. [15] carried out trials on dairy cows in small commercial farms in Kenya using mixed feeds containing *Calliandra* and *S. cannabina*, with conventional diets set as the control. Their results indicated that diets supplemented with *Calliandra* and *S. cannabina* effectively boosted daily milk yield of dairy cows, providing a feasible alternative feeding strategy for small-scale Kenyan farms with considerable economic and environmental benefits.

The rumen is a complex microbial ecosystem in which fibrolytic, amylolytic, and proteolytic microorganisms interact to degrade dietary carbohydrates and proteins and generate volatile fatty acids and microbial protein for the host [16]. Changes in forage source and chemical composition can alter substrate availability and ruminal conditions, thereby reshaping microbial community structure and metabolic activity and influencing fiber degradation, nitrogen metabolism, and nutrient utilization [17,18]. These beneficial responses are presumed to be closely associated with the unique nutritional characteristics of *S. cannabina*. Differences in crude protein concentration, fiber characteristics, and secondary metabolite composition between *S. cannabina* and alfalfa may alter ruminal nitrogen availability and microbial metabolism, thereby affecting nitrogen utilization efficiency [19]. In addition, endogenous bioactive secondary metabolites in *S. cannabina*, including tannins and phenolic compounds, may further modulate ruminal microbial metabolism and maintain relatively stable rumen fermentation conditions, thereby facilitating nutrient digestion and absorption [19,20].

Despite the above findings, existing research on the application of *S. cannabina* in ruminant nutrition remains limited. Most prior investigations only evaluate apparent production performance and basic rumen fermentation indices. Systematic multi-omics datasets describing responses to gradient alfalfa replacement by *S. cannabina* are lacking, especially regarding rumen microecology, microbial–metabolite associations and host phenotypic variation. In addition, appropriate substitution proportions for fattening lambs have not been clearly determined. These knowledge gaps hinder the accurate and standardized use of *S. cannabina* in mutton sheep feeding practice.

Accordingly, we hypothesized that graded replacement of alfalfa with *S. cannabina* would alter rumen microbial community composition and metabolic profiles, leading to changes in rumen fermentation characteristics, digestive enzyme activities, ruminal epithelial morphology, and nutrient digestibility in fattening Aohu hybrid sheep. The objective of this study was to evaluate the effects of different *S. cannabina* substitution levels on growth performance, apparent nutrient digestibility, rumen fermentation, ruminal epithelial morphology, microbial community composition, and metabolite profiles. By integrating animal phenotypic measurements with microbial and metabolomic analyses, this study aimed to provide biological evidence for evaluating *S. cannabina* as a salt-tolerant alternative forage and to inform the selection of appropriate inclusion levels in lamb fattening diets.

## 2. Materials and Methods

### 2.1. Animal Ethics Statement

This animal experiment was reviewed and approved by the Animal Ethics Committee of Tarim University prior to the initiation of the study (Approval No. PA20250326008; approved on 26 March 2025). All animal experimental procedures were conducted in accordance with the approved protocol and relevant regulations for the management and welfare of laboratory animals. A supplementary ethical review for the publication of the research findings was subsequently issued by the Institutional Ethics Committee of Tarim University on 20 April 2026 (Approval No. PB20260420001).

### 2.2. Animals, Experimental Design, and Diets

The experiment was conducted from June to September 2025 at Xiangqing Ecological Farmers’ Professional Breeding Cooperative, Aksu, Xinjiang, China (40°49′2″ N, 80°34′21″ E). A completely randomized design was used. Forty 3-month-old male Aohu hybrid lambs with a mean initial body weight of 22.08 ± 0.31 kg were randomly assigned to five dietary treatments, with eight lambs per treatment. Each lamb was individually housed and served as the experimental unit. The treatments consisted of a control diet containing alfalfa hay without *S. cannabina* hay (CON) and four experimental diets in which alfalfa hay was replaced with *S. cannabina* hay at 25% (SC25), 50% (SC50), 75% (SC75), or 100% (SC100) of the alfalfa component on a dry matter (DM) basis.

All diets were formulated according to the Nutrient Requirements of Meat-Type Sheep and Goats (NY/T 816-2021) [21] and maintained a constant forage-to-concentrate ratio of 50:50 on a DM basis. To minimize potential confounding effects of dietary nutrient composition, the concentrate ingredients were slightly adjusted among treatments to maintain comparable dietary crude protein and metabolizable energy concentrations. Dietary neutral detergent fiber (NDF) and acid detergent fiber (ADF) concentrations were also maintained at comparable levels among treatments. Physically effective neutral detergent fiber (peNDF) was not directly determined in the present study. Thus, although minor adjustments were made to the concentrate formulation for nutrient balancing, the major experimental difference among diets was the progressive replacement of alfalfa hay with *S. cannabina* hay. The ingredient composition and chemical composition of the experimental diets are presented in Table 1, while the detailed nutrient profiles of the diets and the chemical composition of the two principal forage sources, alfalfa hay and *S. cannabina* hay, are provided in Table S1.

The *S. cannabina* used in the present experiment was established in May 2024 at the Tarim University experimental base in Alar, Xinjiang, China, and the first cutting was harvested in October 2024 at the pod-setting stage. At harvest, the forage had a leaf-to-stem ratio of approximately 5.5:1. After harvest, the forage was sun-dried for 2 days, chopped to a theoretical length of approximately 2–3 cm, packed and stored under dry ambient conditions from October 2024 until its use in the feeding trial during June–September 2025. Alfalfa hay, corn straw, and the remaining dietary ingredients were obtained from local commercial suppliers in Xinjiang. The nutrient composition of the principal dietary ingredients was determined before diet formulation and is reported in Table S1.

Lambs were individually housed in partially open pens and had free access to clean drinking water and salt licks throughout the experiment. Experimental diets were offered twice daily at 08:30 and 21:30 h. At the beginning of the trial, the daily DM allowance was estimated at approximately 3% of body weight as an initial feeding reference rather than as a restriction on intake. Feed allowance was subsequently adjusted daily according to individual consumption during the preceding day, with sufficient feed provided to maintain approximately 5–10% refusals. Therefore, animals were allowed ad libitum access to the experimental diets, and feed intake represented voluntary intake rather than a predetermined restricted amount. Feed offered and refusals were recorded to calculate individual daily DM intake.

The experiment consisted of a 10-day adaptation period followed by a 60-day formal feeding period. Environmental temperature and relative humidity were monitored daily at 08:30 and 20:30 throughout the experiment. The corresponding mean temperature and relative humidity recorded at these two monitoring times were 22.6 °C and 65% and 28.7 °C and 52%, respectively.

### 2.3. Data Collection and Sample Analysis

#### 2.3.1. Feed Intake and Growth Performance

Feed offered and refusals were recorded daily for each lamb throughout the experiment to calculate average daily feed intake (ADFI). Lambs were weighed before the morning feeding on d 1, 20, 40, and 60 to determine initial body weight (IBW), final body weight (FBW), average daily gain (ADG), and feed-to-gain ratio (F/G). The calculation formulas were as follows: ADFI = (total feed offered − total feed refused)/number of experimental days; ADG = (FBW − IBW)/number of experimental days; F/G = ADFI/ADG.

#### 2.3.2. Apparent Digestibility

On d 40 of the formal trial, apparent nutrient digestibility was determined using the total fecal collection method. All lambs were individually housed in metabolism cages throughout the digestibility trial to allow for quantitative collection of feces. The digestibility trial consisted of a 7 d adaptation period followed by a 5 d total collection period. Each lamb was fitted with a fecal collection bag, which was checked regularly and replaced as necessary to prevent overflow and sample loss. The metabolism cages were designed to permit separate collection of feces and urine, thereby minimizing urine contamination of fecal samples. In addition, trained personnel visually inspected the cages and fecal collection bags twice daily to verify complete recovery of excreted feces.

Feed offered, refusals, and total fecal output were recorded daily for each lamb. Refusals were collected before the morning feeding, thoroughly mixed, subsampled, and stored at −20 °C until analysis. Total fecal output was weighed daily, and a representative 10% subsample was collected. The fecal subsamples were acidified with 10% sulfuric acid at a ratio of 10:1 (*w*/*v*) and stored at −20 °C. At the end of the collection period, daily fecal subsamples from each animal were pooled proportionally on an individual-animal basis for subsequent chemical analyses.

Subsamples of feed, feed ingredients and refusals were collected. Aliquots of these samples were dried at 105 °C to constant weight to determine DM. For routine chemical analysis, feed and refusal samples, as well as fecal samples, were first dried at 65 °C to constant weight to limit heat-induced degradation of nutrients, then ground. Crude protein (CP) was determined by the Kjeldahl method (AOAC, 2000 [22]; method 976.05), ether extract (EE) by Soxhlet extraction (AOAC, 2005 [23]; method 920.39), and NDF and ADF according to the detergent fiber method [24]. Calcium and phosphorus were determined according to AOAC, 1990 [25], methods 985.35 and 986.24, respectively. Metabolizable energy (ME) was estimated rather than directly measured. Based on the *Nutrient Requirements of Meat-Type Sheep and Goats* (NY/T 816-2021) [21], digestible energy (DE) was estimated from the measured dietary NDF concentration as DE (MJ/kg DM) = 17.211 − 0.135 × NDF (% DM), and ME was subsequently calculated as ME (MJ/kg DM) = 0.046 + 0.820 × DE. Accordingly, ME was estimated as ME = 0.046 + 0.820 × (17.211 − 0.135 × NDF). This equation was applied to the complete experimental diets using their measured NDF concentrations.

#### 2.3.3. Rumen Fermentation Parameters

At the end of the experimental period (d 60), all animals were subjected to a 24 h feed withdrawal before slaughter, following a previously reported pre-slaughter sampling procedure in Hu sheep with modification [26]. Water was withdrawn for 2 h before slaughter. The same fasting procedure was applied to all animals, and rumen digesta samples were collected within 15 min after slaughter. One subsample was subjected to immediate pH measurement using an FE28-standard pH meter (Mettler Toledo, Shanghai, China). The remaining filtrate was centrifuged at 4000× *g* for 15 min at 4 °C using an Eppendorf 5810R centrifuge (Eppendorf AG, Hamburg, Germany), and the resulting supernatant was collected for volatile fatty acid (VFA) and ammonia nitrogen (NH_3_-N) quantification. For VFA profiling, ruminal supernatant was mixed with 25% metaphosphoric acid and analyzed on an Agilent 7890A gas chromatograph (Agilent Technologies Inc., Santa Clara, CA, USA). The concentration of NH_3_-N was measured following the protocol described by Broderick et al. [27]. 

Rumen digesta was also collected for subsequent analyses. Samples for microbial and metabolomic analyses were snap-frozen in liquid nitrogen and stored at −80 °C, whereas samples for digestive enzyme activity determination were stored at −20 °C.

#### 2.3.4. Rumen Digestive Enzyme Activities

The activities of total protease (TPase), α-amylase (α-AMY), cellulase (endo-β-1,4-glucanase, Cel), and hemicellulase (xylanase, HEMI) in rumen fluid were determined using commercial assay kits (Jiangsu Jingmei Biotechnology Co., Ltd., Yancheng, Jiangsu, China) according to the manufacturers’ protocols. TPase activity was determined using azo-casein as the substrate and measured at 366 nm after incubation at 30 °C for 60 min. α-AMY activity was determined by starch hydrolysis after β-amylase inactivation at 70 °C, followed by 3,5-dinitrosalicylic acid (DNS) color development and measurement at 540 nm. Cel and HEMI activities were determined from reducing sugars released from cellulose and xylan, respectively, using the DNS method at 540 nm after incubation for 60 min. Enzyme activities were expressed as azocasein U/(h·mL) for TPase, μg maltose/(min·mL) for α-AMY, μg reducing sugar/(min·mL) for Cel, and nmol xylose/(min·mL) for HEMI. The calculation unit of cellulase activity originally expressed on an hourly basis according to the kit instructions has been revised to a per-minute basis to be consistent with the data presented in the Results section. All enzyme activities were expressed per unit volume of rumen fluid rather than normalized to protein concentration. Intra- and inter-assay coefficients of variation were not provided by the manufacturer and were not independently determined in this study.

#### 2.3.5. Rumen Histomorphology

Immediately after slaughter, the rumen was excised and rinsed with pre-cooled normal saline. A tissue block (approximately 1 cm^2^) was harvested from the ventral sac of the rumen, fixed in 4% paraformaldehyde solution, embedded in paraffin, and serially sectioned. Fields were randomly selected on each section, and intact papillae were chosen following unified observation criteria. Under a 4× objective lens, five intact, fully expanded and appropriately oriented rumen papillae were selected for morphological quantification per lamb. To avoid observer bias, all morphological measurements were performed by an investigator blinded to treatment assignments. Histological slices were digitally scanned using a Pan-noramic SCAN II scanner (Danjie Electronics Co., Ltd., Jinan, China). Morphometric parameters including ruminal papilla length, papilla width and muscular layer thickness were measured using KFSlideOS software (v1.0.5).

#### 2.3.6. Serum Biochemical Parameters

Blood samples were collected from each lamb before the morning feeding on d 1 and d 60. All lambs followed the same fasting procedure prior to both blood samplings to eliminate the interference of fasting duration and feeding rhythm on serum biochemical indices. After standing at room temperature for 40 min, samples were centrifuged at 3000× *g* for 15 min, and serum was collected and stored at −80 °C until analysis. Serum concentrations of total protein (TP), albumin (ALB), glucose (GLU), β-hydroxybutyrate (BHBA), alanine aminotransferase (ALT), and aspartate aminotransferase (AST) were determined using a SpectraMax 190 microplate reader (Molecular Devices, San Jose, CA, USA) with corresponding commercial kits (Jiangsu Jingmei Biotechnology Co., Ltd., Yancheng, Jiangsu, China).

### 2.4. Genomic DNA Extraction and 16S rRNA Gene Sequencing

Total microbial genomic DNA was extracted from rumen samples using the E.Z.N.A. Soil DNA Kit (Omega Bio-tek, Norcross, GA, USA). Eight independent rumen samples from each treatment group (*n* = 8) were used for 16S rRNA sequencing, and samples were not pooled. DNA quality and concentration were assessed by agarose gel electrophoresis and NanoDrop 2000 spectrophotometry (Thermo Scientific, Waltham, MA, USA). The V3–V4 region of the bacterial 16S rRNA gene was amplified using primers 338F and 806R. PCR was conducted in a 20 μL reaction system under the following conditions: 95 °C for 3 min; 27 cycles of 95 °C for 30 s, 55 °C for 30 s, and 72 °C for 30 s; and 72 °C for 10 min. Amplicons were purified, quantified using a Qubit 4.0 fluorometer (Thermo Fisher Scientific, Waltham, MA, USA), and used for library construction with the NEXTFLEX Rapid DNA-Seq Kit. Sequencing was performed on the Illumina NextSeq 2000 platform at Majorbio Bio-Pharm Technology Co., Ltd. (Shanghai, China).

### 2.5. Processing and Analysis of Rumen 16S rRNA Sequencing Data

Raw paired-end reads were quality-filtered using fastp (v0.19.6) and merged using FLASH (v1.2.11). Denoising was performed with the DADA2 plugin within QIIME 2 (v2020.2) to generate amplicon sequence variants (ASVs), and chloroplast and mitochondrial sequences were removed. All samples were rarefied to an equal sequencing depth of 42,336 sequences (the minimum valid read count across all samples) to mitigate sequencing-depth-derived bias. Rarefaction curves verified that this depth sufficiently recovered bacterial community diversity, and corresponding curves are available in the Supplementary Materials. After rarefaction, the average Good’s coverage was 99.09%. Taxonomic assignment was performed via the classify-sklearn Naive Bayes classifier against the SILVA database (v138.2) with a confidence threshold of 0.7. All analyses were implemented on the Majorbio Cloud Platform (www.majorbio.com).

Alpha diversity metrics (Chao1, Shannon, Simpson, ACE, Coverage, and Sobs) were calculated using mothur software (v1.30.1). Wilcoxon rank-sum tests were conducted to evaluate significant differences in alpha diversity between groups. Principal coordinate analysis (PCoA) based on Bray–Curtis dissimilarity coupled with analysis of similarities (ANOSIM) was adopted to investigate inter-group discrepancies in microbial community composition. Linear discriminant analysis effect size (LEfSe) implemented in R (v3.3.1) was performed to identify differentially abundant genera, adopting an all-against-all comparison strategy. Unclassified taxa were excluded, abundance data were normalized, and the taxonomic level was limited to genus. Multiple testing was corrected using the Benjamini–Hochberg (BH) procedure, and genera with an LDA score > 3.0 and an FDR-adjusted *q* < 0.05 were considered differentially abundant.

### 2.6. Metabolomic Profiling of Rumen Contents

Rumen content samples were thawed on ice, and approximately 100 mg of each sample was accurately weighed into a 2 mL centrifuge tube. A total of 800 μL of methanol/water solution (4:1, *v*/*v*) containing internal standards was added to each sample. After cryogenic grinding at 50 Hz for 6 min at −10 °C, samples were subjected to ultrasonic extraction for 30 min at 4 °C, followed by incubation at −20 °C for 30 min to precipitate proteins. The mixture was then centrifuged at 13,000× *g* for 15 min at 4 °C, and the supernatant was collected and transferred into autosampler vials for subsequent LC-MS analysis.

Quality control (QC) samples were prepared by pooling equal volumes of supernatant from all experimental samples to evaluate the stability and reproducibility of the LC–MS analytical system. QC samples were injected at regular intervals (approximately every 5–15 analytical samples) throughout the analytical sequence. QC-based RSD distribution analysis was used to assess analytical precision. In the positive-ion dataset, 83.72% of metabolic features in the raw QC data and 85.39% after data preprocessing exhibited an RSD ≤ 30%. For the combined QC dataset, 81.05% and 81.73% of metabolic features in the raw and preprocessed data, respectively, exhibited an RSD ≤ 30%. Metabolic features with an RSD > 30% across QC samples were excluded from downstream statistical analysis. The corresponding RSD distribution plots, including those for the negative-ion dataset, together with the available QC chromatographic and instrument-stability assessments, are provided in the Supplementary Materials.

Untargeted metabolomic profiling was performed on an ultra-high-performance liquid chromatography coupled with Orbitrap Exploris 240 mass spectrometry system (UHPLC-Orbitrap Exploris 240; Thermo Fisher Scientific, Waltham, MA, USA) equipped with an ACQUITY UPLC HSS T3 column (100 mm × 2.1 mm, 1.8 μm; Waters, Milford, MA, USA). The mobile phase consisted of solvent A (95% water + 5% acetonitrile with 0.1% formic acid) and solvent B (47.5% acetonitrile + 47.5% isopropanol + 5% water with 0.1% formic acid). Gradient elution was carried out at a flow rate of 0.40 mL/min, with an injection volume of 10 μL, and the column temperature was maintained at 45 °C. Mass spectrometric detection was operated in both positive and negative electrospray ionization (ESI) modes, with a mass scan range of *m*/*z* 50–1200.

### 2.7. Processing and Analysis of Metabolomic Data

Raw LC-MS data were imported into Progenesis QI software (v3.0; Waters Corporation, Milford, MA, USA) for baseline correction, peak detection, retention time alignment, and peak area normalization. Metabolite annotation was conducted by matching primary MS and MS/MS spectra against public databases including the Human Metabolome Database (HMDB), METLIN, and the Majorbio in-house metabolite library, with a mass tolerance set below 10 ppm. The resulting data matrix was uploaded to the Majorbio Cloud Platform for downstream statistical and bioinformatic analysis.

A strict quality filtering workflow was applied to ensure data reliability. First, metabolic features detected in fewer than 80% of samples within any group were removed. Missing values were imputed with the minimum intensity value of the corresponding feature. Peak intensities were then subjected to total-sum normalization to reduce systematic bias across samples. Features with an RSD > 30% across all QC samples were excluded from further analysis. The retained data were log_10_-transformed and scaled via Pareto scaling to eliminate dimensional differences between metabolites and ensure equal contribution of all variables to model construction.

Unsupervised principal component analysis (PCA) was first performed to visualize the overall metabolic variation and natural clustering patterns among dietary groups. Subsequently, supervised partial least squares discriminant analysis (PLS-DA) was conducted using the *ropls* package (v1.6.2) in R to maximize inter-group separation and identify metabolites driving sample discrimination. Model robustness was evaluated via seven-fold cross-validation, yielding an R^2^Y (goodness of fit) of 0.891 and a Q^2^ (predictive ability) of 0.725, indicating reliable model performance. Permutation testing with 200 random permutations of class labels was further applied to exclude model overfitting; the Q^2^ intercept from permutation tests was −0.712 (below zero), confirming no overfitting and statistical significance of the grouping discrimination. Additionally, non-parametric analysis of similarities (ANOSIM) was performed to verify that inter-group metabolic differences were significantly greater than intra-group variation, supporting the biological validity of sample grouping.

Differential metabolites between dietary groups were screened based on two combined criteria: (1) variable importance in projection (VIP) value > 1 derived from the PLS-DA model; (2) false discovery rate (FDR)-adjusted *q* < 0.05 from univariate statistical tests to correct for multiple comparisons. Finally, all identified differential metabolites were mapped to the Kyoto Encyclopedia of Genes and Genomes (KEGG) database for pathway annotation and functional enrichment analysis.

### 2.8. Statistical Analysis

Statistical analyses were performed using SPSS 27.0 software (IBM Corp., Armonk, NY, USA). Figures for serum biochemical parameters were generated using GraphPad Prism 8.3 software (GraphPad Software, San Diego, CA, USA).

Prior to statistical analysis, the normality of data distribution was verified using the Shapiro–Wilk test, and homogeneity of variance was assessed using Levene’s test. For parameters including growth performance, apparent nutrient digestibility, rumen fermentation parameters, ruminal enzyme activities, and rumen histomorphological indices, one-way analysis of variance (one-way ANOVA) was applied to compare differences among dietary treatment groups when the assumptions of normal distribution and homogeneity of variance were met. When a significant treatment effect was detected (*p* < 0.05), Tukey’s honestly significant difference (Tukey’s HSD) test was used for post hoc multiple comparisons among groups. For data with a normal distribution but unequal variances, Welch’s ANOVA was used for overall difference testing, followed by Tamhane’s T2 test for post hoc multiple comparisons. Because maintaining iso-nitrogenous and iso-energetic diets required concurrent adjustment of other concentrate ingredients across the graded replacement levels (e.g., soybean meal from 3.20% to 7.70%), the treatments did not constitute an isolated single-factor gradient. Therefore, dietary treatment was analyzed as a categorical factor, orthogonal polynomial contrasts were not applied, and the results were interpreted as differences among treatment groups rather than as formal dose–response trends.

Serum biochemical parameters were measured on Day 1 and Day 60 of the experiment. All animals were fed the same basal diet before the formal trial, and one-way ANOVA confirmed no significant differences in baseline values of serum biochemical parameters among groups on Day 1 (*p* > 0.05). Analysis of covariance (ANCOVA) was used to evaluate the effects of different dietary treatments on serum biochemical parameters on Day 60, with the measured values of corresponding indices on Day 60 as the dependent variable, dietary treatment as the fixed factor, and the baseline values of corresponding indices on Day 1 as the covariate. Prior to ANCOVA, the linear relationship between the covariate and the dependent variable, as well as the interaction between the covariate and dietary treatment, was examined to verify the assumption of homogeneity of regression slopes. When a significant dietary treatment effect was observed (*p* < 0.05), pairwise comparisons among groups were performed based on the ANCOVA-adjusted estimated marginal means, with Bonferroni correction for multiple comparisons.

In the figures of serum biochemical parameters, raw mean ± standard error of the mean (mean ± SEM) values of each treatment group on Day 60 are presented, while the labeled significant differences among groups and corresponding *p* values are determined based on ANCOVA results with Day 1 baseline values as covariates. All other data are expressed as mean ± SEM. Significant differences among groups are marked with different lowercase letters, and *p* < 0.05 was considered statistically significant.

## 3. Results

### 3.1. Feed Intake and Growth Performance

The effects of replacing alfalfa with different proportions of *S. cannabina* hay on feed intake and growth performance of lambs are shown in Table 2. The ADFI of lambs in the SC75 group was significantly higher than that in the CON, SC25, SC50, and SC100 groups (*p* < 0.05). No significant differences were observed in FBW, ADG, or F/G among the groups (*p* > 0.05).

### 3.2. Apparent Digestibility

The effects of replacing alfalfa with different proportions of *S. cannabina* hay on apparent nutrient digestibility of lambs are shown in Table 3. The CP digestibility in the SC25 group was significantly lower than that in the SC50 and SC75 groups (*p* < 0.05). The EE digestibility in the SC50, SC75, and SC100 groups was significantly higher than that in the SC25 group (*p* < 0.05), whereas the CON group showed no significant difference from either group. The NDF digestibility in the SC50 and SC100 groups was significantly higher than that in the SC25 group (*p* < 0.05). The ADF digestibility in the SC100 group was significantly higher than that in the CON and SC25 groups (*p* < 0.05). No significant difference was observed in DM digestibility among the groups (*p* = 0.823).

### 3.3. Rumen Digestive Enzyme Activities and Rumen Histomorphology

**Figure 1:**
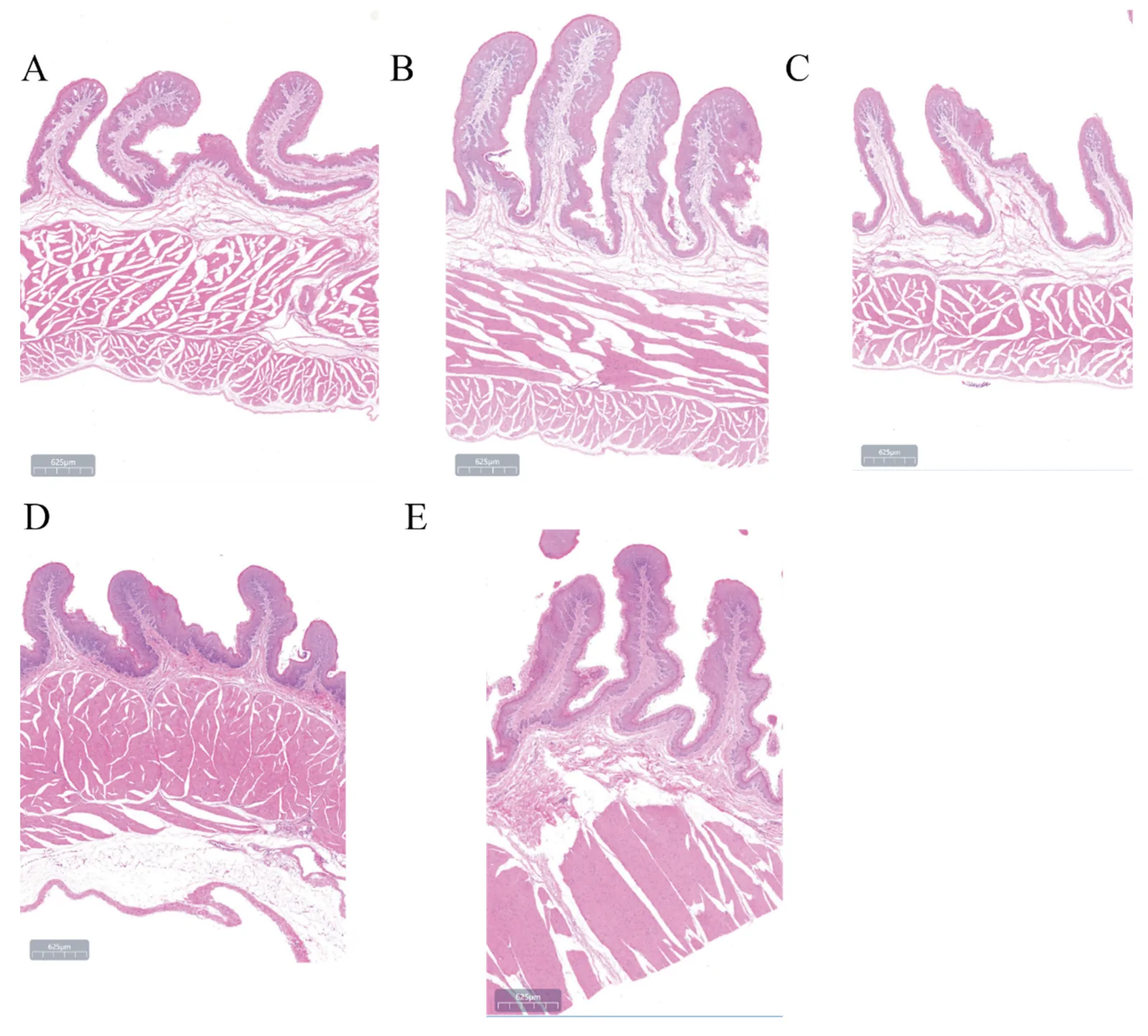
Effects of replacing alfalfa with *Sesbania cannabina* hay on ruminal tissue morphology in lambs. CON group: (**A**) 4.0×; SC25 group: (**B**) 4.0×; SC50 group: (**C**) 4.0×; SC75 group: (**D**) 4.0×; and SC100 group: (**E**) 4.0×. Scale bar = 625 μm.

The effects of replacing alfalfa with different proportions of *S. cannabina* hay on ruminal digestive enzyme activities and rumen histomorphology of lambs are shown in Table 4 and Figure 1. The activities of total protease, α-amylase, cellulase, and hemicellulase were significantly affected by dietary treatment (*p* < 0.001). Total protease activity in the CON and SC75 groups was significantly higher than that in the SC25, SC50, and SC100 groups (*p* < 0.001). The α-amylase activity in the SC75 group was significantly higher than that in the other groups (*p* < 0.001), whereas no significant differences were observed between the CON group and the other replacement groups (*p* > 0.05). Cellulase activity in the SC50 and SC100 groups was significantly higher than that in the CON, SC25, and SC75 groups (*p* < 0.001). Hemicellulase activity in the SC75 and SC100 groups was significantly higher than that in the CON, SC25, and SC50 groups (*p* < 0.001).

The effects of dietary treatment on rumen histomorphological parameters are shown in Figure 1. The ruminal muscular layer thickness in the SC75 and SC100 groups was significantly higher than that in the CON group (*p* = 0.046), whereas the SC25 and SC50 groups showed no significant difference from either group. No significant differences were observed in rumen papilla length or rumen papilla width among the groups (*p* > 0.05).

### 3.4. Rumen Fermentation Parameters

The effects of replacing alfalfa with different proportions of *S. cannabina* hay on rumen fermentation parameters of lambs are shown in Table 5. The ruminal pH in the SC75 group was significantly lower than that in the CON, SC25, SC50, and SC100 groups (*p* < 0.001), whereas no significant differences were observed among the CON, SC25, and SC50 groups (*p* > 0.05). The concentrations of TVFA, acetate, propionate, and butyrate in the SC50, SC75, and SC100 groups were significantly higher than those in the CON and SC25 groups (*p* < 0.001), and these concentrations in the SC75 group were significantly higher than those in the SC50 and SC100 groups (*p* < 0.001). The acetate-to-propionate ratio in the CON group was significantly higher than that in all replacement groups (*p* < 0.001). The NH_3_-N concentration in the SC50 group was significantly higher than that in the other groups (*p* < 0.001), whereas that in the SC100 group was significantly lower than that in the CON, SC25, and SC75 groups (*p* < 0.001).

### 3.5. Serum Biochemical Parameters

**Figure 2:**
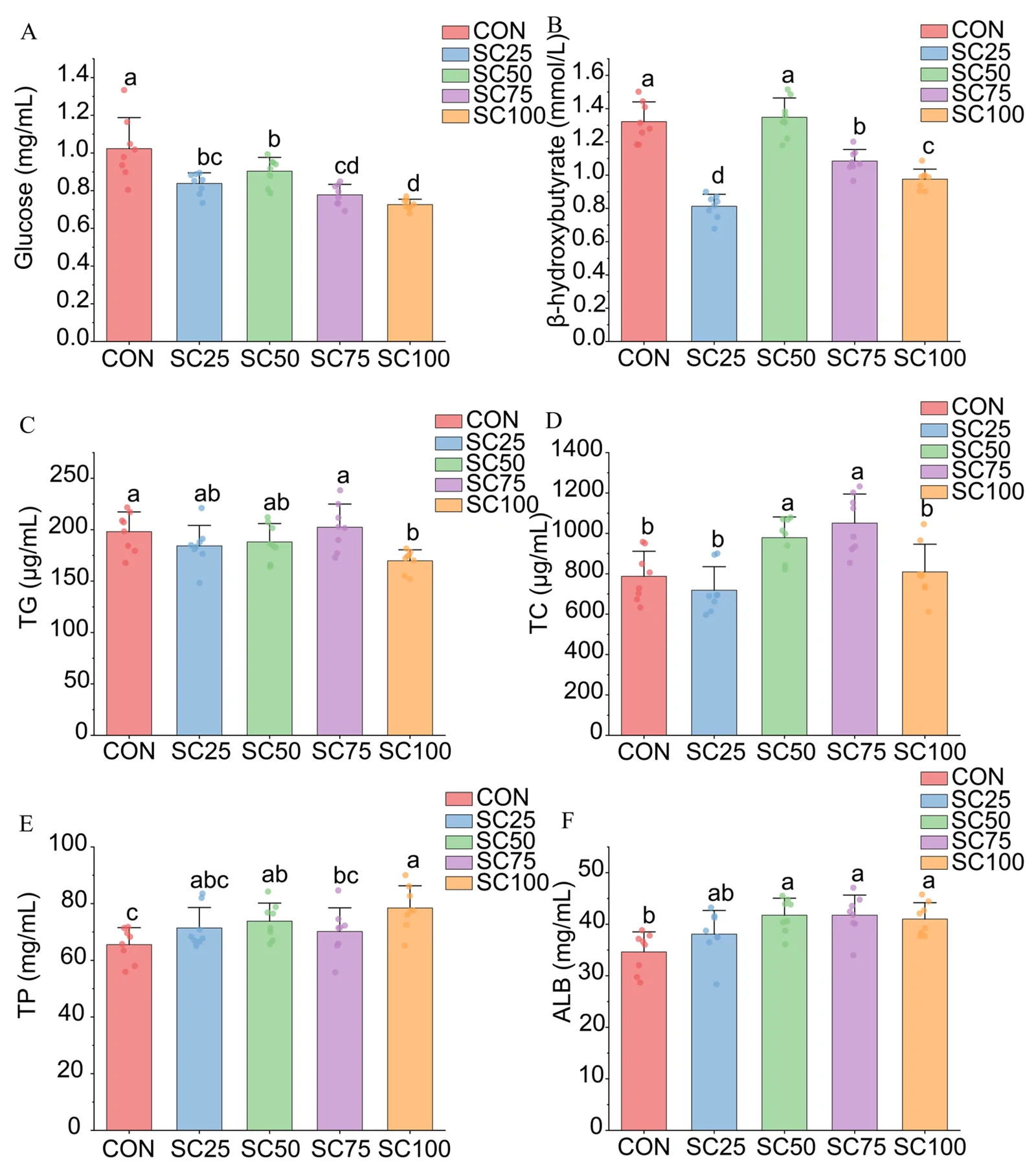
Effects of replacing alfalfa with *Sesbania cannabina* at different proportions on serum biochemical parameters in lambs. (**A**) TP; (**B**) ALB; (**C**) GLU; (**D**) BHBA; (**E**) ALT; and (**F**) AST. Bars represent group means, error bars indicate the standard error of the mean (SEM), and dots represent individual values. Different lowercase letters indicate significant differences among treatment groups for the same parameter (*p* < 0.05), whereas the same or shared letters indicate no significant difference (*p* > 0.05).

The effects of replacing alfalfa with different proportions of *S. cannabina* hay on serum biochemical parameters of lambs are shown in Figure 2. No significant differences were observed in serum biochemical indices among the groups on d 1 (*p* > 0.05; Figure S1). On d 60, the serum GLU concentrations in the SC25, SC50, SC75, and SC100 groups were significantly lower than that in the CON group (*p* < 0.05). The serum BHBA concentration in the SC25 group was significantly lower than that in the other groups (*p* < 0.05), whereas the CON and SC50 groups showed significantly higher values than the remaining treatment groups (*p* < 0.05). The serum ALT concentration in the CON group was significantly higher than that in all *S. cannabina* replacement groups (*p* < 0.05), while no significant difference was observed in AST concentration among the groups (*p* > 0.05). The serum TP concentration in the SC50 and SC100 groups was significantly higher than that in the CON group (*p* < 0.05). The serum ALB concentration in the SC50, SC75, and SC100 groups was significantly higher than that in the CON group (*p* < 0.05).

### 3.6. Rumen Bacterial Community Structure

**Figure 3:**
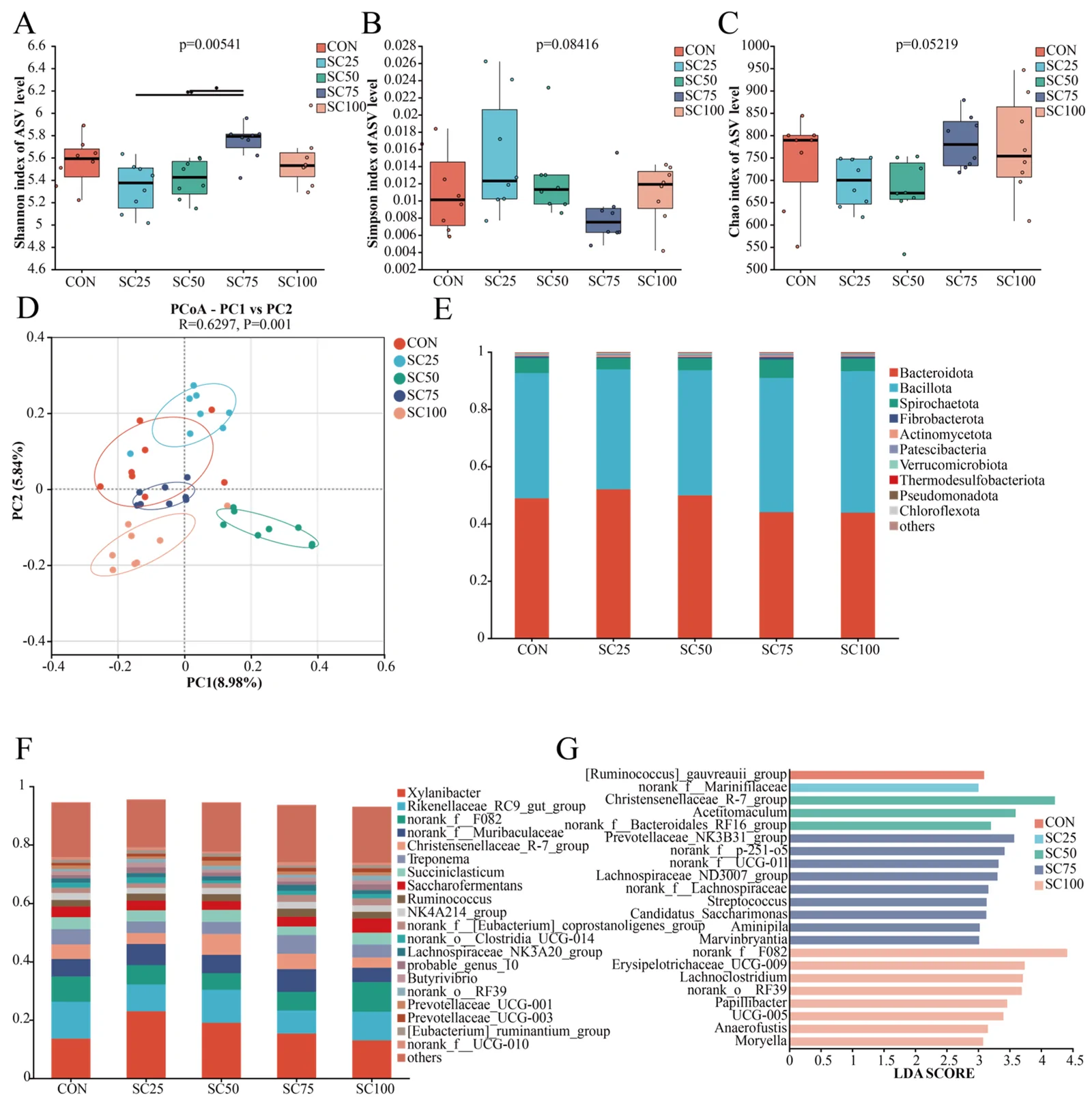
Effects of replacing alfalfa with *Sesbania cannabina* at different proportions on alpha diversity, community structure, and differential taxa of the rumen microbiota in lambs. (**A**) Shannon index; (**B**) Simpson index; (**C**) Chao1 index; (**D**) principal coordinates analysis (PCoA) based on Bray–Curtis distances showing differences in rumen bacterial community structure among the treatment groups; (**E**) relative abundance composition at the phylum level; (**F**) relative abundance composition at the genus level; (**G**) differential genera identified by LEfSe analysis. * *p* < 0.05, ** *p* < 0.01.

After rarefaction to 42,336 sequences per sample, 10,059 ASVs were obtained (Table S2). Alpha diversity analysis showed significant differences in the Shannon index among groups (*p* = 0.005; Figure 3A–C), with SC75 being higher than SC25 and SC50. The Simpson index did not differ among groups (*p* > 0.05), whereas the Chao1 index showed a marginal difference (*p* = 0.052), with relatively lower richness in SC25 and SC50 and higher richness in SC75 and CON.

PCoA based on Bray–Curtis distances showed a clear separation among groups (Figure 3D), with PC1 and PC2 explaining 8.98% and 5.84% of the variation, respectively. ANOSIM further confirmed significant differences in rumen bacterial community structure among treatments (R = 0.6297, *p* = 0.001).

At the phylum level (Figure 3E), *Bacteroidota*, *Bacillota*, and *Spirochaetota* were dominant, accounting for more than 95% of total sequences. *Bacteroidota* abundance was lower in SC75 and SC100 than in CON, SC25, and SC50 (*p* = 0.038; Table S3), whereas *Bacillota* increased with increasing replacement level. Accordingly, the *Bacillota*/*Bacteroidota* ratio was higher in SC75 and SC100 than in CON (*p* < 0.05). At the genus level (Figure 3F), *Xylanibacter*, *Rikenellaceae_RC9_gut_group*, and *norank_f__F082* were dominant, and *Xylanibacter* tended to be more abundant in SC25 and SC50.

LEfSe analysis was further performed to identify differential genera among the treatment groups (LDA > 3.0, *p* < 0.05; Figure 3G). The results showed that *norank_f_F082* had the highest LDA score in the SC100 group, and its relative abundance differed significantly among the treatment groups (*p* < 0.05). In addition, *Erysipelotrichaceae_UCG-009*, *Lachnoclostridium*, *Papillibacter*, and *UCG-005* were also significantly more abundant in the SC100 group than in the other treatment groups (*p* < 0.05). Notably, *norank_f_UCG-011*, *Prevotellaceae_NK3B31_group*, *norank_f_Lachnospiraceae*, *norank_f_p-251-o5*, and *Lachnospiraceae_ND3007_group* were all significantly more abundant in the SC75 group than in the CON group and the other *S. cannabina* replacement groups (*p* < 0.05), and all had LDA scores greater than 3, with *Prevotellaceae_NK3B31_group* showing an LDA score above 3.5. Moreover, *Christensenellaceae_R-7_group* and *Acetitomaculum* were significantly more abundant in the SC50 group than in the CON group and the other treatment groups (*p* < 0.05), with *Christensenellaceae_R-7_group* showing an LDA score greater than 4. On the other hand, *[Ruminococcus]_gauvreauii_group* and *norank_f_Marinifilaceae* were among the main enriched taxa in the CON and SC25 groups, respectively, and their relative abundances differed significantly among the treatment groups (*p* < 0.05).

### 3.7. Identification and Differential Analysis of Rumen Metabolites

#### 3.7.1. Rumen Metabolic Profiles and Inter-Group Difference Testing

**Figure 4:**
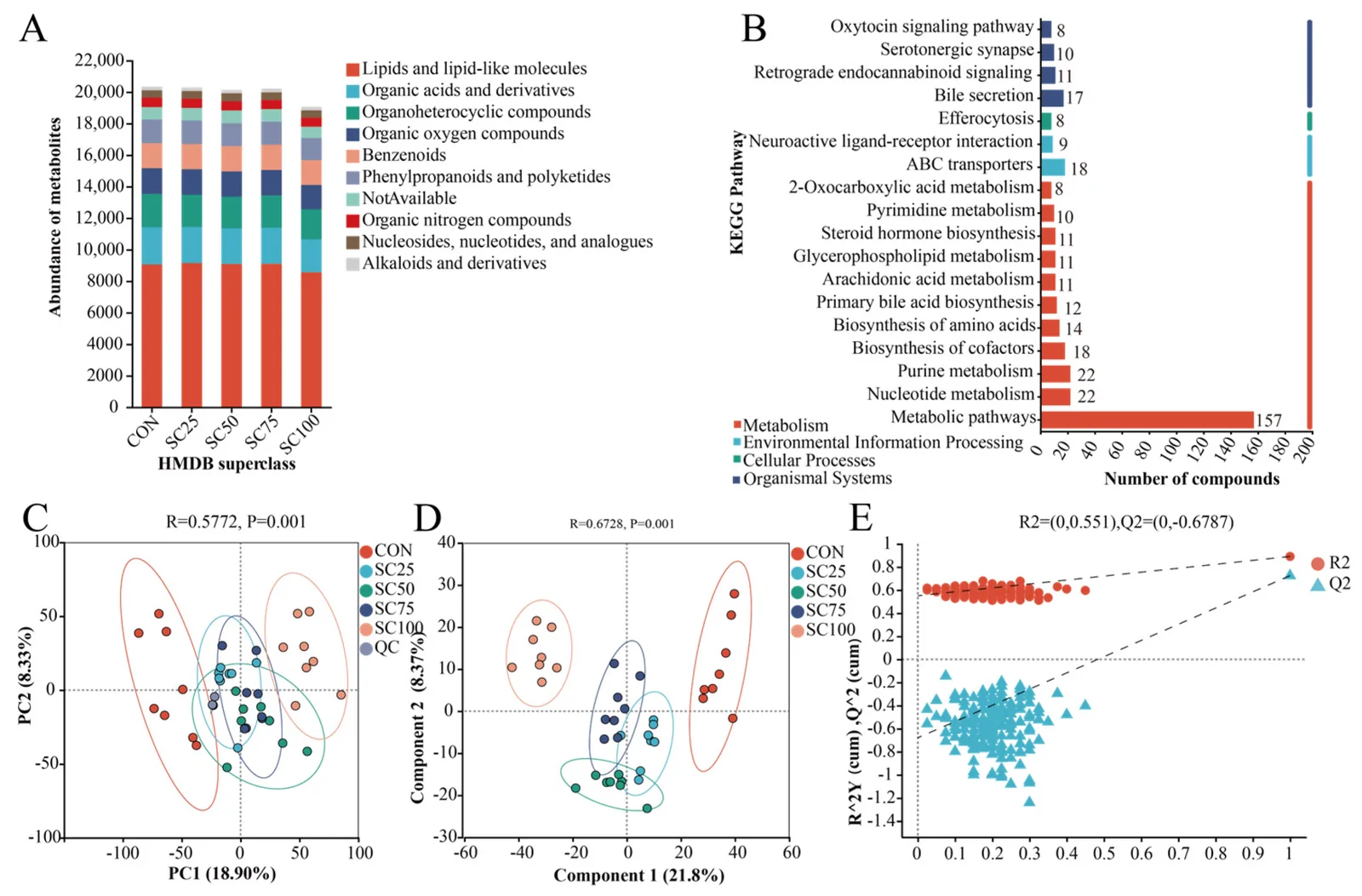
Overall overview of rumen metabolic profiles and inter-group difference analyses in lambs under different levels of *Sesbania cannabina* replacement. (**A**) Stacked bar chart of HMDB metabolite classifications. (**B**) KEGG pathway enrichment analysis. (**C**) PCA score plot. (**D**) PLS-DA score plot. (**E**) Validation of the PLS-DA model.

Untargeted metabolomics identified 1733 metabolites in rumen fluid samples (Table S4). HMDB classification showed that metabolites were mainly lipids and lipid-like molecules, followed by organic acids and derivatives and organoheterocyclic compounds (Figure 4A). Overall metabolite composition was similar among groups, although lipid-related metabolites tended to decrease in the high-replacement groups, especially SC100.

KEGG annotation showed that most metabolites were assigned to metabolic pathways, particularly nucleotide/purine metabolism and cofactor biosynthesis (Figure 4B). PCA showed a separation trend among groups, with PC1 and PC2 explaining 18.90% and 8.33% of the variance, respectively (Figure 4C). ANOSIM confirmed significant inter-group differences (*p* = 0.001), and QC samples clustered tightly, indicating good analytical stability. PLS-DA further improved group discrimination (Component 1 = 21.8%, Component 2 = 8.37%; Figure 4D), and permutation testing supported model robustness (R^2^ intercept = 0.551, Q^2^ intercept = −0.6787; Figure 4E).

#### 3.7.2. Screening of Differential Metabolites and Identification of Key Biomarkers

**Figure 5:**
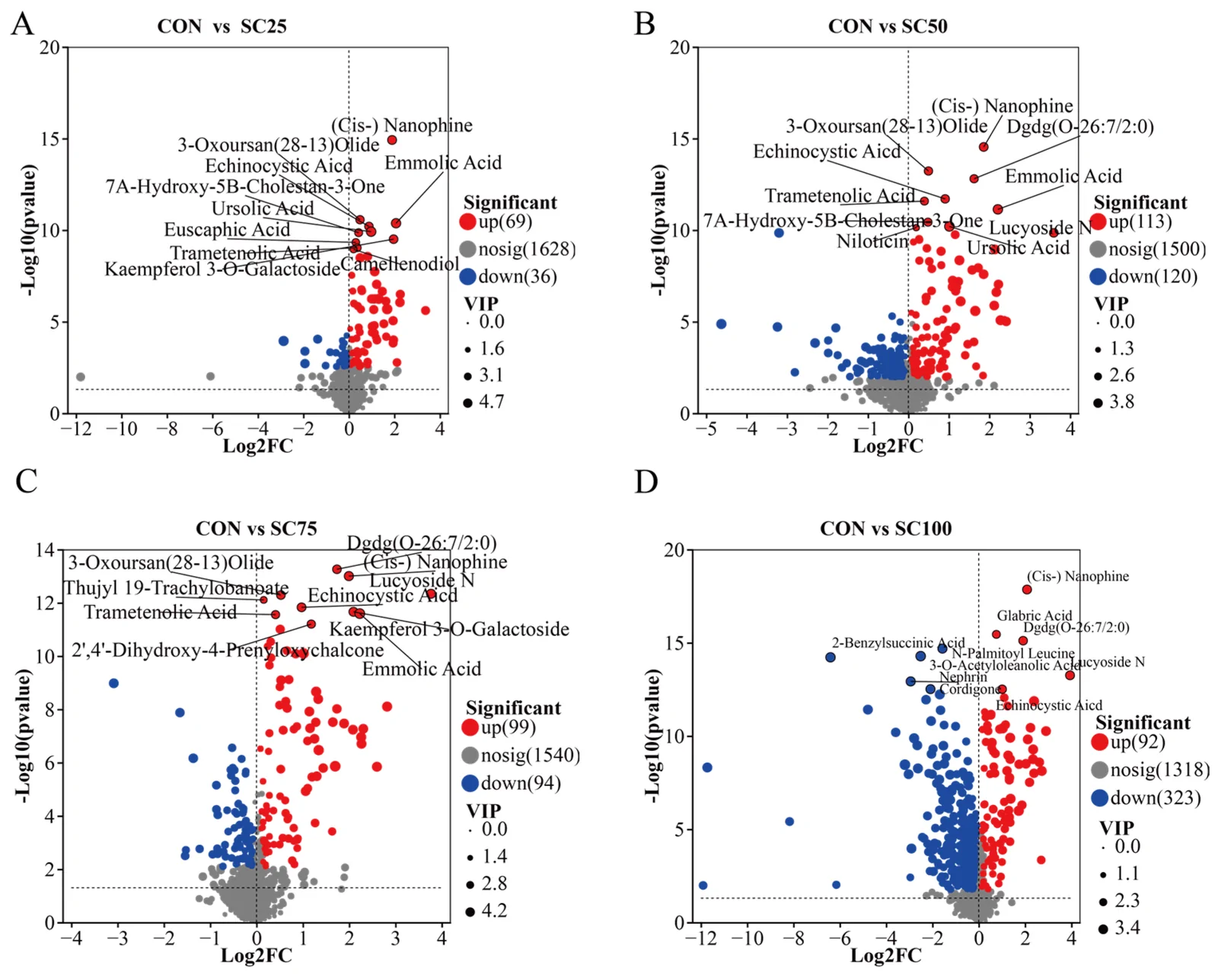
Effects of replacing alfalfa with *Sesbania cannabina* at different proportions on ruminal metabolic profiles and inter-group differences in lambs. (**A**) CON vs. SC25; (**B**) CON vs. SC50; (**C**) CON vs. SC75; and (**D**) CON vs. SC100. The *x*-axis represents log2(fold change), and the *y*-axis represents −log10(*p* value). Red dots indicate significantly upregulated metabolites, blue dots indicate significantly downregulated metabolites, and gray dots indicate metabolites with no significant difference. Dot size represents the VIP value.

To evaluate the effects of replacing alfalfa with *S. cannabina* at different proportions on the ruminal metabolic profiles of lambs, differential metabolites were screened using PLS-DA combined with univariate statistical analysis, with the criteria defined as VIP > 1.0 and q < 0.05 after FDR correction. The results showed that, compared with the CON group, all *S. cannabina* substitution groups exhibited metabolic alterations to varying degrees. A total of 105 differential metabolites were identified in the SC25 group, including 69 upregulated and 36 downregulated metabolites (Figure 5A). In the SC50 and SC75 groups, the numbers and directions of differential metabolites were relatively balanced, with 113 upregulated and 120 downregulated metabolites detected in the SC50 group, and 99 upregulated and 94 downregulated metabolites in the SC75 group, respectively (Figure 5B,C). Notably, the SC100 group showed the most pronounced metabolic alterations, with a total of 415 differential metabolites identified, including 323 downregulated and 92 upregulated metabolites (Figure 5D), indicating that a high proportion of *S. cannabina* replacement induced a broader metabolic shift characterized predominantly by downregulation.

Several triterpenoids and lipid-related metabolites, including (Cis-) Nanophine, Echinocystic acid, Emmolic acid, and Trametenolic acid, were repeatedly detected across comparisons and may serve as key markers of *Sesbania cannabina* substitution. DGDG (O-26:7/2:0) also differed significantly in SC50 and SC75, indicating marked effects on lipid and glycolipid metabolism at medium-to-high replacement levels.

**Figure 6:**
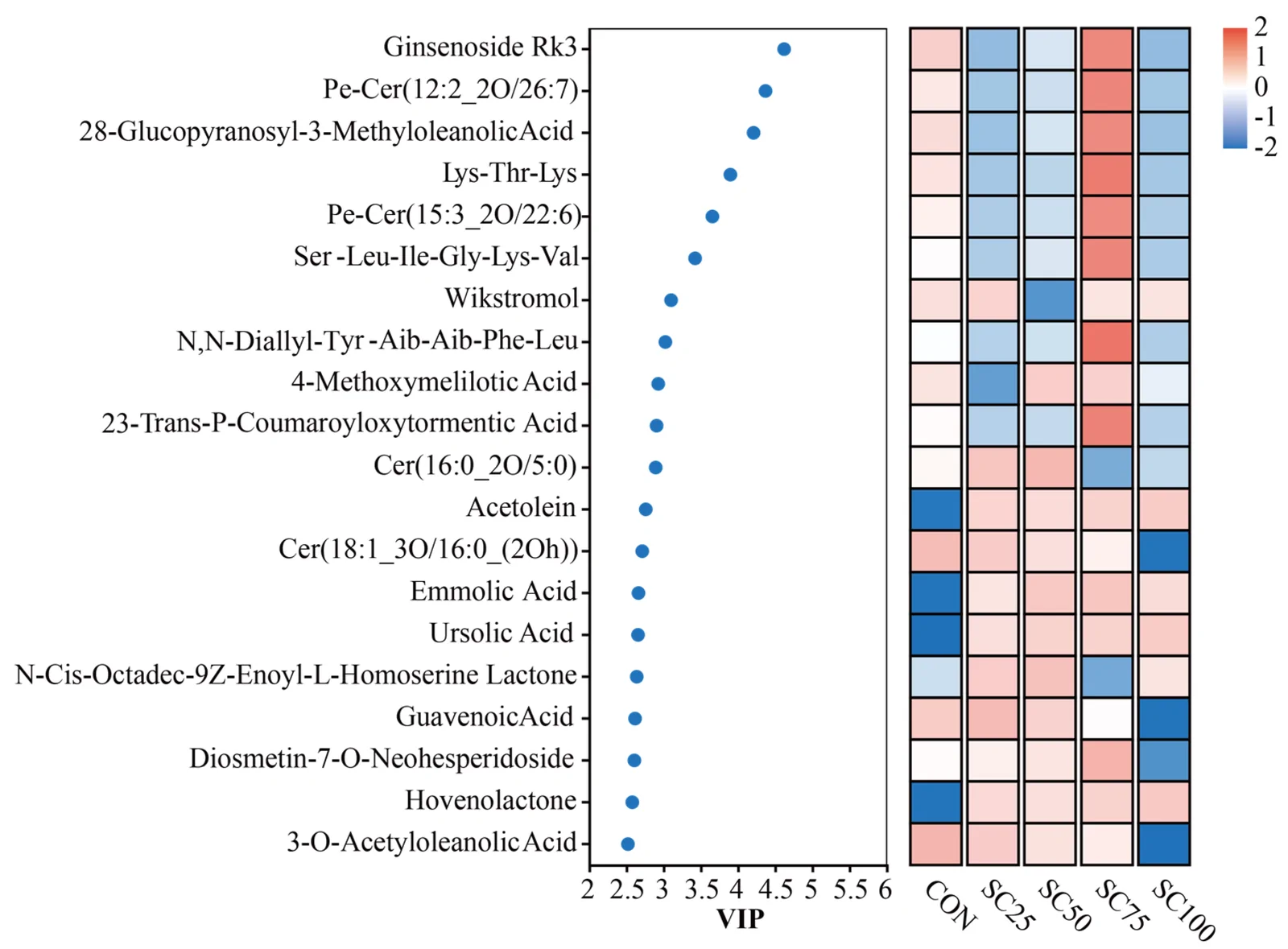
Top 20 differential metabolites ranked by VIP values and their abundance patterns among treatment groups. The left panel shows the variable importance in projection (VIP) scores of the top 20 metabolites identified by PLS-DA, all of which had VIP values > 2.5. The right panel shows a hierarchical clustering heatmap of the same metabolites across the CON, SC25, SC50, SC75, and SC100 groups. Red indicates relatively high abundance, whereas blue indicates relatively low abundance.

The top 20 metabolites ranked by VIP all had values > 2.5 (Figure 6), indicating strong contributions to group discrimination. These metabolites mainly included Ginsenoside Rk3, Pe-Cer/Cer-related lipids, 28-Glucopyranosyl-3-Methyloleanolic Acid, Lys-Thr-Lys, and Wikstromol, representing triterpenoids, sphingolipids/ceramides, peptides, and phenolic derivatives. Heatmap clustering showed clear group-specific patterns: Ginsenoside Rk3, some Pe-Cer-related metabolites, and 28-Glucopyranosyl-3-Methyloleanolic Acid were more abundant in SC75 but lower in SC25 and SC100, whereas Ursolic Acid and Emmolic Acid generally increased after *Sesbania cannabina* replacement. These results indicate that the ruminal metabolic profiles differed among replacement levels, with SC75 exhibiting a distinct metabolite pattern.

#### 3.7.3. KEGG Pathway Enrichment of Differential Metabolites

**Figure 7:**
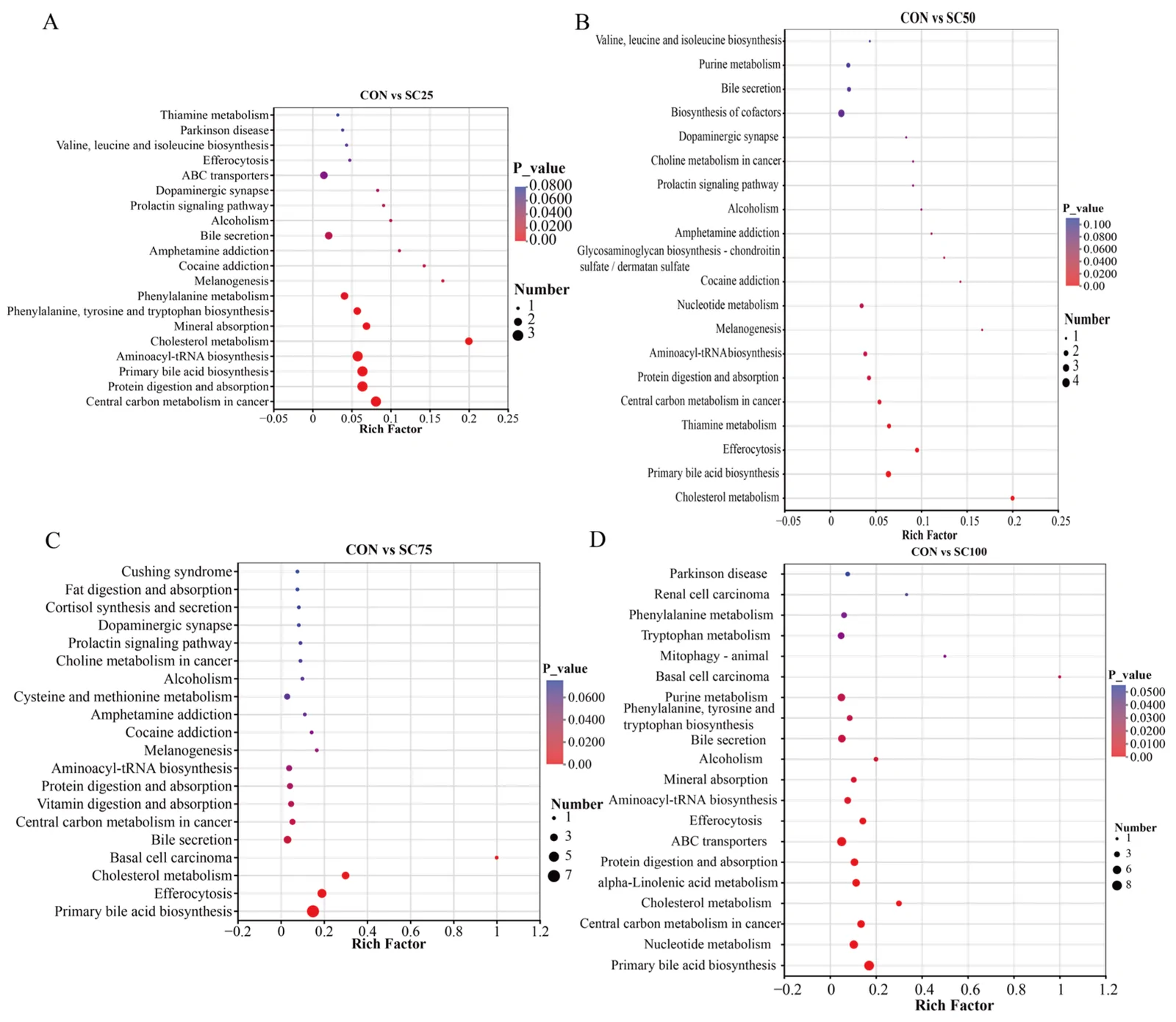
Effects of replacing alfalfa with *Sesbania cannabina* at different proportions on ruminal metabolic pathway enrichment profiles and inter-group differences in lambs. (**A**) CON vs. SC25; (**B**) CON vs. SC50; (**C**) CON vs. SC75; and (**D**) CON vs. SC100. Differential metabolites were subjected to KEGG pathway enrichment analysis. The *x*-axis represents the rich factor, and the *y*-axis shows the significantly enriched pathways. Bubble size indicates the number of enriched differential metabolites within each pathway, and bubble color indicates the significance level of pathway enrichment.

KEGG enrichment analysis showed that *Sesbania cannabina* replacement significantly altered multiple metabolic pathways (Figure 7A–D). With increasing replacement level, the number of enriched pathways, significance levels, and matched metabolites generally increased. Differential metabolites were mainly enriched in pathways related to bile acid and cholesterol metabolism, including primary bile acid biosynthesis, cholesterol metabolism, and bile secretion, as well as aminoacyl-tRNA biosynthesis and protein digestion and absorption. Primary bile acid biosynthesis and cholesterol metabolism were most significantly enriched in SC75 and SC100, suggesting that the metabolic effects of *Sesbania cannabina* were mainly associated with lipid, bile acid, and amino acid metabolism.

### 3.8. Correlation Analysis of Rumen Bacteria, Metabolites, and Lamb Phenotypes

**Figure 8:**
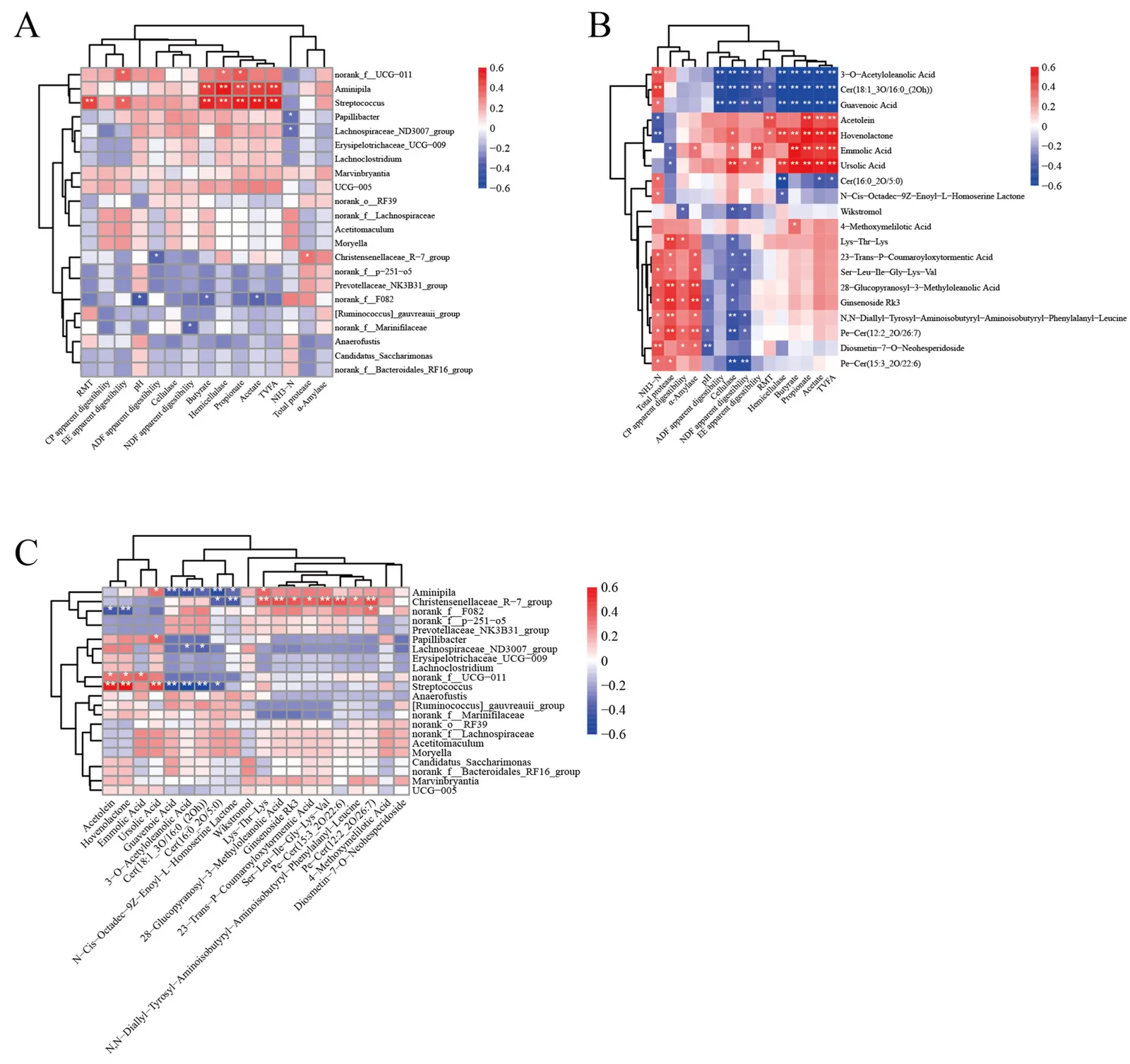
Correlation heatmaps between differential rumen bacterial genera, metabolites, and phenotypic indicators. (**A**) Correlation analysis between differential bacterial genera and phenotypic indicators; (**B**) correlation analysis between differential metabolites and phenotypic indicators; (**C**) correlation analysis between differential bacterial genera and differential metabolites. Red indicates a positive correlation, while blue indicates a negative correlation. * *p* < 0.05, ** *p* < 0.01.

Spearman’s correlation analysis revealed significant associations among differential genera, rumen fermentation traits, and nutrient digestibility (Figure 8A). *Aminipila* and *Streptococcus* showed similar positive correlation patterns and were positively correlated with acetate, propionate, butyrate, and TVFA concentrations (*p* < 0.01). *Streptococcus* was also positively correlated with ruminal muscular thickness and apparent EE digestibility, whereas *norank_f_UCG-011* was positively correlated with EE digestibility as well as acetate and propionate concentrations (*p* < 0.05). In contrast, *Papillibacter* and *Lachnospiraceae_ND3007_group* were negatively correlated with total protease activity, while *Christensenellaceae_R-7_group* was negatively correlated with apparent NDF digestibility but positively correlated with total protease activity (*p* < 0.05). *norank_f__F082* was negatively correlated with ruminal pH, acetate, and butyrate, and *norank_f__Marinifilaceae* was negatively correlated with butyrate concentration (*p* < 0.05).

The metabolite–phenotype correlation heatmap (Figure 8B) also showed clear functional clustering. Acetolein, Hovenolactone, Emmolic Acid, and Ursolic Acid were positively correlated with TVFA and its major components, as well as hemicellulase activity and EE digestibility (*p* < 0.05), whereas 3-O-Acetyloleanolic Acid, Cer(18:1_3O/16:0_(2Oh)), and Guavenoic Acid were negatively correlated with cellulase activity, NDF and ADF digestibility, and VFA production (*p* < 0.01). Another cluster represented by Lys-Thr-Lys, Ser-Leu-Ile-Gly-Lys-Val, Ginsenoside Rk3, and Diosmetin-7-O-Neohesperidoside was positively correlated with NH_3_-N, total protease activity, CP digestibility, and α-amylase activity but negatively correlated with cellulase activity and fiber digestibility (*p* < 0.05).

Correlations between differential genera and metabolites (Figure 8C) further indicated that *Christensenellaceae_R-7_group* was associated with the largest number of metabolites, showing positive correlations with several oligopeptides, Ginsenoside Rk3, and ceramide derivatives (*p* < 0.01). *Streptococcus* and *norank_f_UCG-011* were positively correlated with Acetolein, Hovenolactone, and Ursolic Acid, but negatively correlated with Guavenoic Acid and sphingolipid-related metabolites, whereas *norank_f__F082* showed an opposite pattern. Overall, these results suggest that *Streptococcus* and *Aminipila* were associated with enhanced ruminal VFA production, whereas *Christensenellaceae_R-7_group* was more closely related to protein degradation and peptide metabolism.

## 4. Discussion

### 4.1. Growth Performance, Apparent Digestibility, and Ruminal Enzyme Activities

Growth performance provides an integrative indication of dietary adequacy in growing ruminants [28]. In the present study, replacing alfalfa with *S. cannabina* did not significantly affect FBW, ADG, or F/G, consistent with previous findings [12]. The *S. cannabina* hay contained 15.0% CP, lower than alfalfa (18.0%) and the previously reported 22–24%, likely because it was harvested at the pod-setting stage, when increased maturity and fiber deposition reduce CP concentration. Differences in cultivar, environment, and processing may also contribute. To maintain comparable dietary CP concentrations across treatments, the proportion of soybean meal was increased from 3.20% to 7.70% as the proportion of *S. cannabina* increased. Therefore, the observed responses should be interpreted as the combined effects of *S. cannabina* inclusion and corresponding concentrate adjustment, representing the nutritional response to an integrated feeding strategy rather than a forage substitution effect alone. Notably, although the digestibility of several nutrient fractions changed, growth performance remained unaffected, indicating that changes in digestibility did not necessarily translate into improved productive performance. Conversely, the maintenance of growth performance after dietary reformulation suggests that the overall feeding strategy adequately met the nutritional requirements of growing lambs. This discrepancy between digestive responses and growth performance may be attributed to the relatively short experimental period, compensatory nutrient utilization, or differences in the partitioning of absorbed nutrients between maintenance and tissue deposition. Overall, these findings support the practical feasibility of incorporating *S. cannabina* into nutritionally balanced diets with appropriate concentrate adjustment.

Apparent nutrient digestibility reflects the proportion of ingested nutrients that is not recovered in feces and is influenced by multiple factors, including feed chemical composition, ruminal degradation, passage rate, and post-ruminal digestion [29]. In the present study, apparent DM digestibility did not differ significantly among treatments, and this finding was consistent with the results reported by Farghaly et al. [30], suggesting that substitution of alfalfa with *S. cannabina* did not substantially alter overall dietary DM utilization. In contrast, significant treatment effects were detected for CP, EE, NDF, and ADF digestibility, indicating that *S. cannabina* inclusion was associated with changes in the utilization of specific nutrient fractions rather than a general enhancement of digestive capacity.

CP digestibility was lower in SC25 than in SC50 and SC75. One possible explanation is that differences in forage protein characteristics and their interactions with the rumen microbial community may have altered the extent or site of protein degradation. Previous studies have suggested that shifts in dietary protein sources can modify microbial substrate utilization and nitrogen metabolism [31,32]. However, microbial adaptation to *S. cannabina* protein was not directly evaluated in the present study; therefore, this explanation remains hypothetical. Differences between alfalfa and *S. cannabina* in protein degradability, fiber-bound protein, or secondary metabolites may also have contributed to the observed variation in CP digestibility and warrant further investigation.

The greater EE digestibility observed in SC50–SC100 than in SC25, together with the higher NDF digestibility in SC50 and SC100 and higher ADF digestibility in SC100 than in CON and SC25, suggests that *S. cannabina* inclusion was associated with changes in the utilization of lipid and structural carbohydrate fractions. Comparable responses have been reported in previous studies evaluating alternative forage resources. For example, one study found that diets containing *S. cannabina* or reed were associated with greater digestible crude protein (DCP) and higher NDF and ADF digestibility than an alfalfa-based control diet [30]. Although DCP and apparent CP digestibility are not directly equivalent measures of protein utilization, these findings provide additional evidence that alternative forage sources may differ from alfalfa in their protein and fiber utilization characteristics. However, the responses varied among replacement levels and therefore should not be interpreted as evidence that increasing *S. cannabina* inclusion progressively improved digestive efficiency. Differences in fiber composition, lignification, leaf-to-stem ratio, protein characteristics, and concentrations of plant secondary metabolites between alfalfa and *S. cannabina* may partly account for the observed responses. In particular, tannins, saponins, phenolics, and other secondary metabolites may influence ruminal protein degradation and fiber digestion in a concentration-dependent manner [33]. Because these compounds were not quantitatively characterized in the forage used in the present study, their contribution to the observed digestibility responses remains uncertain.

Changes in ruminal enzyme activities showed some correspondence with the variation in apparent nutrient digestibility. For example, relatively high total protease activity in SC75 coincided with higher CP digestibility, whereas increased cellulase and hemicellulase activities in some *S. cannabina*-containing treatments were accompanied by greater fiber digestibility. Previous studies have reported associations between ruminal hydrolytic enzyme activities and the degradation of corresponding substrates [34,35]. Nevertheless, enzyme activities measured in rumen digesta represent only a snapshot of potential enzymatic activity and do not necessarily reflect whole-tract in vivo digestive capacity. Thus, the present findings should be interpreted as associative rather than causal evidence, suggesting that changes in the ruminal enzymatic environment accompanied, rather than necessarily caused, differences in nutrient utilization.

Similarly, α-amylase activity was highest in SC75. This observation may indicate differences among treatments in the ruminal enzymatic environment related to starch and readily fermentable carbohydrate degradation [36]. However, because starch disappearance, microbial enzyme expression, and carbohydrate metabolic fluxes were not directly measured, the biological significance of this difference cannot be fully established. In particular, it cannot be concluded that the higher α-amylase activity directly caused subsequent changes in rumen fermentation. Further studies integrating enzyme activity measurements with microbial functional genes, substrate-specific degradation, and metabolic flux analyses would help clarify these relationships.

### 4.2. Ruminal Fermentation Parameters and Tissue Morphology

Volatile fatty acids (VFAs) represent a major form of energy supply in ruminants and generally contribute approximately 70% of the energy required for maintenance and production, thereby playing an important role in feed utilization efficiency [37]. In the present study, replacement with *S. cannabina* significantly increased the concentrations of total VFA (TVFA), acetate, propionate, and butyrate, while the acetate-to-propionate (A:P) ratio decreased with increasing replacement level, indicating a directional shift in the ruminal fermentation pattern. Previous studies have shown that propionate is a key substrate for gluconeogenesis in ruminants and accounts for approximately 60% of whole-body glucose utilization. A lower A:P ratio is generally associated with greater energy utilization efficiency [38,39], and a reduction in the acetate-to-propionate ratio has also been reported to be more favorable for growth in sheep [40,41].

This shift toward a more propionate-oriented fermentation pattern may have partly compensated for the lower feed intake observed in the SC100 group by helping to maintain energy supply, thereby contributing to growth performance comparable to that of the control group. The significantly higher acetate concentration in the SC75 group may have been associated with improved fiber digestibility. Overall, the changes in VFA concentrations observed in this study may indicate that lambs receiving *S. cannabina* were able to ferment dietary substrates more extensively and consequently generate greater amounts of VFAs, potentially increasing the energy available to the host [42].

In addition, ruminal VFA concentrations were generally higher in the *S. cannabina* groups, while ruminal pH was also significantly higher than that in the CON group, although values in all groups remained within the physiological range of 6.9–7.3. During passive absorption of VFAs across the ruminal epithelium, H^+^ can be removed from the ruminal lumen. Once VFAs enter epithelial cells and dissociate in the cytoplasm, however, the released H^+^ must be extruded to maintain intracellular pH homeostasis. Monocarboxylate transporters and Na^+^/H^+^ exchangers involved in intracellular pH regulation can transport H^+^ back into the ruminal lumen or extracellular space [43,44]. These mechanisms may contribute to maintaining a relatively stable ruminal pH despite changes in VFA production and absorption. Nevertheless, the present ruminal pH results should be interpreted with caution because pH was not monitored continuously and therefore does not represent the dynamic diurnal pH profile of the rumen.

The ruminal NH_3_-N results further indicated that ruminal nitrogen metabolism did not change synchronously with VFA production. Ruminal NH_3_-N concentrations ranged from 17.67 to 30.22 mg/dL across treatments. In combination with the differences in the ruminal bacterial community among replacement treatments, these changes suggest that replacing alfalfa with *S. cannabina* altered the balance among dietary protein degradation, ammonia release, and microbial ammonia assimilation [45]. The relatively high NH_3_-N concentration in the SC50 group may be attributed to more intensive proteolysis and deamination in the rumen, together with insufficient microbial capture and utilization of ammonia. In contrast, the lower NH_3_-N level in the SC100 group may indicate reduced ammonia production or greater efficiency of microbial nitrogen incorporation. Notably, the SC75 group exhibited higher bacterial diversity and significant enrichment of core taxa such as the *Prevotellaceae_NK3B31_group* and *Streptococcus*, suggesting a more coordinated fermentation pattern that may have improved the synchronization of energy supply and nitrogen utilization for microbial growth.

Compared with ruminal fermentation parameters, the effects of *S. cannabina* replacement on ruminal tissue morphology were less pronounced but still showed a clear pattern. Ruminal papilla length and width did not differ significantly among groups, whereas ruminal muscular thickness was significantly greater in the SC75 and SC100 groups than in the CON group. Previous studies have demonstrated that ruminal fermentation end products, especially propionate and butyrate, can regulate the growth and development of ruminal epithelial and muscular tissues [46,47]. Therefore, the increased ruminal muscular thickness observed in these two groups may reflect adaptive tissue remodeling under sustained high fermentative activity. This morphological change was selective rather than extensive and did not indicate structural damage to the rumen, further supporting the view that *S. cannabina* replacement optimized ruminal physiological and metabolic function without impairing normal ruminal tissue structure. 

### 4.3. Serum Biochemical Parameters

Serum biochemical indices can, to some extent, reflect the nutritional status, metabolic state, and overall health of animals, and they are commonly used to evaluate the effects of dietary changes on systemic metabolism [48].

Serum TP and ALB concentrations were higher than those of the control group in some *S. cannabina* treatments. As TP and ALB are associated with protein nutritional status, hepatic protein synthesis, and whole-body protein turnover [48,49], these changes may reflect alterations in protein metabolism following *S. cannabina* replacement. This interpretation is partly consistent with the higher apparent crude protein digestibility observed in some treatment groups. However, because TP and ALB are also influenced by hepatic synthesis, protein degradation, fluid balance, and nutrient intake, these results alone cannot demonstrate enhanced protein synthesis or nitrogen retention.

AST and ALT are intracellular transaminases that catalyze the transfer of amino groups between amino acids and keto acids. They are mainly distributed within cells of the liver and other tissues, and their activities in the circulation are normally relatively low. When hepatocytes are damaged or when cell membrane permeability or cellular integrity is compromised, these intracellular enzymes may be released into the bloodstream, resulting in increased serum AST and ALT activities [50]. In the present study, serum ALT activity was generally lower in the *S. cannabina* replacement groups, whereas AST did not differ significantly among treatments and remained within an appropriate physiological range. Therefore, no obvious biochemical evidence of hepatocellular injury was observed under the present experimental conditions. Nevertheless, AST and ALT alone are insufficient to establish hepatoprotective effects or complete dietary safety.

Energy-related indices showed a more complex response. Serum GLU was generally lower in the *S. cannabina* groups, whereas BHBA concentrations differed among replacement treatments. This pattern was not fully consistent with the increase in ruminal propionate, an important gluconeogenic precursor in ruminants [51]. The lack of a parallel increase in circulating GLU suggests that changes in ruminal propionate availability do not necessarily translate directly into higher blood glucose concentrations. Blood glucose homeostasis is also regulated by hepatic gluconeogenesis, tissue glucose uptake, hormonal regulation, nutrient partitioning, and whole-body energy requirements [51]. Therefore, the lower GLU concentration should not be interpreted simply as evidence of inadequate energy supply.

Plant secondary metabolites may provide another possible explanation. *S. cannabina*, as a leguminous plant, may contain saponins, flavonoids, phenolic compounds, and other bioactive constituents that have been associated with glucose metabolism in previous studies [52]. However, these compounds were not quantified in the experimental diets, and gluconeogenic enzyme activities or glucose metabolic fluxes were not measured. Their contribution to the reduced serum GLU concentration therefore remains speculative.

The variation in BHBA should also be interpreted cautiously because circulating BHBA is influenced by ruminal butyrate metabolism, hepatic fatty acid oxidation, lipid mobilization, and peripheral ketone utilization. Thus, the lower BHBA concentration in SC25 or the relatively higher value in SC50 cannot be attributed to a single metabolic pathway based on the present data. 

Overall, the responses of serum metabolites were not completely parallel to those of ruminal fermentation and nutrient digestibility. Although some *S. cannabina* treatments showed higher protein digestibility, VFA concentrations, TP, and ALB, these changes did not result in significant improvements in average daily gain or feed efficiency. This indicates that changes in digestive and metabolic indices do not necessarily translate directly into improved growth performance, probably because absorbed nutrients are partitioned among maintenance, tissue deposition, and other physiological processes. Collectively, the present results suggest that replacing alfalfa with *S. cannabina* induced adjustments in protein and energy metabolism without obvious adverse effects, rather than providing evidence for a generalized enhancement of metabolic efficiency. Further studies combining nitrogen balance, plant secondary metabolite characterization, and targeted metabolic measurements are required to clarify the underlying mechanisms.

### 4.4. Effects of S. cannabina Replacing Alfalfa on Ruminal Microbiota Diversity and Community Composition

The ruminal microbial community is central to the degradation and fermentation of plant-derived feedstuffs in ruminants, and changes in its composition can influence nutrient utilization efficiency and fermentation characteristics [53,54]. At the microbial diversity level, the response induced by 75% dietary replacement was mainly reflected in changes in community diversity and evenness rather than a substantial increase in species richness. Greater microbial diversity may facilitate the utilization of a broader range of dietary substrates and expand the functional potential of the microbial community [39,55]. The SC75 group exhibited a higher Shannon diversity index together with relatively high feed intake and a pronounced fermentation response, suggesting that, under the conditions of the present study, this replacement level supported a more diverse ruminal microbial community.

At the phylum level, Bacteroidota and Bacillota were the dominant bacterial phyla, consistent with the typical ruminal microbial composition reported previously in ruminants [56,57]. Members of Bacteroidota are capable of producing a wide range of carbohydrate-active enzymes and are extensively involved in the degradation of complex plant polysaccharides [58,59,60], whereas many Bacillota taxa participate in the hydrolysis and fermentation of structural carbohydrates and other macromolecular substrates [61]. As the proportion of *S. cannabina* replacement increased, the relative abundance of Bacillota increased, whereas that of Bacteroidota decreased, with these changes being particularly evident in the SC75 and 100% *S. cannabina* replacement (SC100) groups. These shifts may represent adaptive responses of the ruminal microbiota to differences between *S. cannabina* and alfalfa in cell-wall characteristics, substrate availability, and secondary metabolite composition [62,63].

Accordingly, the Bacillota/Bacteroidota ratio increased in the SC75 and SC100 groups. Some studies in ruminants have reported associations between changes in this ratio and nutrient digestibility, fermentation characteristics, or productive performance [64,65,66]. However, its biological significance is highly dependent on experimental conditions, and findings are not consistent across different ruminant production systems. Therefore, an increased Bacillota/Bacteroidota ratio should not be regarded as a universal indicator of improved rumen function or animal performance. The present results also support this interpretation, as the SC100 group exhibited the highest ratio without a corresponding improvement in growth performance, while its metabolomic profile was characterized predominantly by downregulated metabolites. Taken together, the phylum-level shifts observed in this study are more appropriately interpreted as adaptive microbial responses to dietary ingredient replacement rather than as evidence of intrinsically improved productive efficiency.

At the genus level, *Xylanibacter* is commonly detected at relatively high abundance in ruminants and has been associated with fiber degradation and host digestive function [35,67,68]. Consistent with previous observations, *Xylanibacter* was among the dominant genera across all treatment groups in the present study and remained relatively abundant in the SC25 and SC50 groups. These findings suggest that low to moderate levels of *S. cannabina* replacement did not markedly disrupt the major fiber-associated microbial community. However, the lower protein digestibility observed in SC25 indicates that microbial adaptation at a low replacement level may not have equally promoted the utilization of all nutrient fractions.

*Christensenellaceae_R-7_group* was significantly enriched in the SC50 group and was positively correlated with total protease activity but negatively correlated with apparent NDF digestibility. Previous studies have reported associations of this taxon with dietary nutrient composition, ruminal health, and host metabolic status [69,70]. Nevertheless, its metabolic functions have not yet been fully characterized. Therefore, these correlations cannot be considered direct evidence that *Christensenellaceae_R-7_group* regulates protein metabolism. Its enrichment may instead reflect microbial adaptation to the nutrient environment of the SC50 diet, and its specific function requires further validation.

In the SC75 group, *Prevotellaceae_NK3B31_group* was significantly enriched and showed a relatively high LDA score. *Prevotella* is an abundant and metabolically versatile genus in the rumen and is capable of utilizing hemicellulose, pectin, other non-cellulosic polysaccharides, and proteinaceous substrates [62,71]. Its abundance is also responsive to changes in dietary composition. However, *Prevotellaceae_NK3B31_group* cannot be assumed to possess all of the metabolic functions reported for well-characterized *Prevotella* species. Thus, its enrichment in SC75 does not provide direct evidence that it promoted propionate production or polysaccharide degradation. Together with changes in *norank_f_UCG-011*, *norank_f_Lachnospiraceae*, *norank_f_p-251-o5*, and *Lachnospiraceae_ND3007_group*, the enrichment of this taxon more likely reflects an overall microbial adaptation to the SC75 diet. Lachnospiraceae-related taxa are generally involved in the fermentation of complex carbon substrates and the production of VFAs [72,73], although their specific contributions to the fermentation pattern observed in the present study remain to be verified.

In contrast, *norank_f_F082* was enriched in the SC100 group and exhibited the highest LDA score. Previous studies have reported associations between F082-related taxa and ruminal fermentation characteristics [74], but the functional properties of this group remain poorly understood. Its negative correlations with ruminal pH, acetate, and butyrate indicate statistical associations only and do not establish a specific metabolic function. Considering the predominantly downregulated metabolomic profile and the lack of improvement in growth performance in SC100, the pronounced microbial restructuring induced by complete replacement with *S. cannabina* is more likely to represent adaptation to a substantially altered dietary environment rather than the establishment of a more metabolically efficient microbial system.

Overall, replacement of alfalfa with *S. cannabina* induced distinct changes in the ruminal bacterial community among treatment groups at the genus level. The SC75 group exhibited higher microbial diversity together with distinct fermentation characteristics, whereas SC100 showed pronounced microbial restructuring without a corresponding improvement in animal growth performance. Importantly, the relationships observed among microbial taxa, fermentation parameters, enzyme activities, and metabolites are associative and do not establish causality. Therefore, the functional roles of the differential microbial taxa identified in the present study require further validation. Future studies integrating metagenomic and transcriptomic analyses with targeted metabolite measurements and functional validation are needed to clarify the contributions of these taxa to ruminal fermentation and nutrient utilization.

### 4.5. Effects of S. cannabina Replacing Alfalfa on the Ruminal Metabolome

Ruminal metabolites represent the downstream biochemical expression of interactions among dietary composition, microbial metabolism, and host-associated processes [16,75]. In the present study, untargeted metabolomics putatively annotated 1733 metabolites, predominantly comprising lipids and lipid-like molecules, organic acids and derivatives, and organoheterocyclic compounds. These metabolite annotations were considered consistent with MSI level 2 because they were supported by MS and MS/MS spectral matching against public and in-house databases but were not confirmed using authentic reference standards under identical analytical conditions. The broad chemical diversity of the annotated metabolites is consistent with the complex biochemical environment of the rumen, in which dietary substrates undergo extensive microbial transformation and generate a wide range of metabolic products [76]. KEGG annotation further indicated that many of the annotated metabolites were associated with the broad functional category of “Metabolism”, reflecting the metabolic complexity of the ruminal ecosystem [77]. At the multivariate level, PCA and PLS-DA revealed separation among treatment groups, suggesting that dietary replacement of alfalfa with *S. cannabina* was associated with shifts in the overall ruminal metabolic profile.

Pathway enrichment analysis provided additional evidence for differences in metabolic profiles among replacement treatments. Differential metabolites were repeatedly mapped to primary bile acid biosynthesis, cholesterol metabolism, bile secretion, aminoacyl-tRNA biosynthesis and protein digestion and absorption pathways, with broader enrichment generally observed in SC75 and SC100 than in SC25 and SC50. These results indicate that higher replacement levels influenced a wider range of lipid- and amino acid-associated metabolic processes. Notably, the recurrent enrichment of bile acid- and cholesterol-related metabolite sets highlights steroid- and lipid-associated metabolism as an important component of the response to *S. cannabina* replacement. Consistent with this interpretation, in vivo microbial bile acid intervention has been shown to modulate amino acid and lipid metabolism through core secondary bile acids and their downstream metabolites [78]. Thus, alterations in bile acid-associated metabolic networks may be linked to the coordinated remodeling of lipid and amino acid metabolism observed in the present study, although targeted analyses are required to determine whether these pathways are directly involved. 

Nevertheless, these KEGG results should be interpreted cautiously. Pathway enrichment in untargeted ruminal metabolomics is based on structural annotation and database mapping and therefore does not necessarily indicate that the complete canonical pathway is operating within the rumen. This consideration is particularly important for pathways such as primary bile acid biosynthesis and bile secretion, which are conventionally associated with host tissues. Thus, the present findings are more appropriately interpreted as evidence that metabolites structurally or functionally associated with bile acid, sterol, and lipid metabolism were altered by *S. cannabina* replacement. Given the important roles of sterol- and bile acid-related compounds in lipid interactions, membrane properties, and microbial responses to environmental stressors [79,80], these changes may reflect modification of the chemical environment to which ruminal microorganisms were exposed.

Several triterpenoid- and lipid-related metabolites contributed strongly to the discrimination among treatments, as indicated by their relatively high VIP values and fold changes. These included (Cis-)-Nanophine, Echinocystic acid, Emmolic acid, and Trametenolic acid. Triterpenoids are synthesized through pathways originating from acetyl-CoA and, in many biological systems, share metabolic precursors with lipid biosynthesis. Previous work on Yarrowia lipolytica, for example, showed that increasing acetyl-CoA availability could simultaneously enhance squalene and fatty acid production [81]. Although such observations cannot be directly extrapolated to the rumen microbial community, they provide a biochemical basis for considering potential interactions between triterpenoid-associated metabolism and cellular lipid metabolism [82,83]. Therefore, the changes in triterpenoid-like metabolites observed here may reflect altered dietary input, microbial biotransformation, or both, and targeted analyses will be required to determine their origin and functional consequences.

DGDG (O-26:7/2:0) also differed significantly in the SC50 and SC75 groups. DGDG is a glycolipid associated with membrane structure and lipid organization in multiple biological systems [84]. Its differential abundance, together with enrichment of cholesterol metabolism and ABC transporter-related pathways, suggests that moderate-to-high *S. cannabina* replacement may influence metabolites associated with membrane lipid composition and transport processes. However, because the detected DGDG and related lipid molecules may originate from dietary material, microorganisms, or subsequent microbial transformation, their source cannot be conclusively determined from untargeted metabolomic data alone.

VIP-based screening further showed that several of the most discriminatory metabolites belonged to sphingolipid/ceramide-related lipid classes and triterpenoid- or saponin-like compounds. PE-Cer/Cer-related molecules are associated with membrane lipid organization, cellular signaling, and stress responses [85]. Their differential abundance among treatments therefore suggests that membrane-associated lipid metabolism may be sensitive to *S. cannabina* replacement. Similarly, marked changes in metabolites putatively annotated as Ginsenoside Rk3, Ursolic acid, Emmolic acid, and 28-Glucopyranosyl-3-Methyloleanolic acid indicate substantial variation in triterpenoid- and saponin-associated metabolism. These changes may reflect the direct introduction of plant secondary metabolites, microbial transformation of dietary compounds, or interactions between these processes. The occurrence of these triterpenoid- and saponin-like metabolites is also consistent with the known phytochemical characteristics of the genus *Sesbania*, which has been reported to contain saponins, terpenoids, and phenolic compounds. In the congeneric species *Sesbania grandiflora*, these phytochemical classes have been associated with a shift in in vitro rumen fermentation toward higher total VFA and propionate production [86], broadly resembling the fermentation pattern observed in the present study. In addition, variation in small peptide metabolites such as Lys-Thr-Lys is consistent with alterations in peptide turnover and nitrogen-related metabolism, in agreement with the enrichment of aminoacyl-tRNA biosynthesis and protein digestion and absorption pathways.

The heatmap further demonstrated that the abundance patterns of these discriminatory metabolites varied among replacement levels. SC75 was characterized by relatively high abundances of several high-VIP metabolites, including Ginsenoside Rk3, selected PE-Cer-related molecules, and 28-Glucopyranosyl-3-Methyloleanolic acid, whereas these metabolites did not show a corresponding further increase in SC100. Instead, SC100 exhibited a broader pattern of metabolite reduction and greater overall divergence from CON. Collectively, these findings indicate that SC75 and SC100 represented qualitatively different metabolic states. SC75 was associated with selective increases in several lipid- and triterpenoid-related metabolites, whereas complete replacement resulted in more extensive metabolic reorganization.

Overall, the metabolomic results demonstrate that the ruminal metabolic environment differed among *S. cannabina* replacement treatments, with prominent changes involving lipid-, sterol-, triterpenoid-, and amino acid-related metabolites. Moderate-to-high replacement levels, particularly SC75, produced distinct changes in several membrane lipid- and secondary metabolite-associated compounds, whereas complete replacement generated the greatest overall departure from the control metabolic profile. These observations indicate distinct metabolic responses among *S. cannabina* replacement treatments. Nevertheless, because the present untargeted metabolomic analysis primarily provides relative abundance and annotation-based information, the biological origins and functional roles of the key metabolites should be confirmed by targeted quantification and, where possible, integrated analyses of rumen fermentation, microbial composition, and animal performance.

### 4.6. Correlation Analysis of Rumen Bacteria, Metabolites, and Lamb Phenotypes

The correlation analysis provides an integrative view of how microbial shifts, metabolite changes, and host phenotypes were connected across replacement levels. Although correlation does not establish causality, the association patterns observed here help clarify the functional organization of the ruminal response to *S. cannabina* replacement.

In this study, *Aminipila* and *Streptococcus* were significantly positively correlated with acetate, propionate, butyrate, and TVFA, suggesting that these two taxa were associated with a highly active fermentation state. As an important genus related to carbohydrate and starch degradation, *Streptococcus* is often associated with rapid fermentation of non-structural carbohydrates when enriched [87]. Its positive correlation with VFA accumulation is therefore biologically plausible. Notably, Streptococcus was also positively correlated with apparent EE digestibility and ruminal muscular thickness. Combined with the higher VFA levels and increased ruminal muscular thickness observed in the SC75 and SC100 groups, these findings indicate that *Streptococcus* and *Aminipila* are more likely important accompanying taxa under conditions of elevated fermentation activity rather than sole determinants of specific phenotypic changes. This also suggests that the enhanced fermentation induced by *S. cannabina* replacement was more likely the result of overall microbial network reconstruction than the action of any single genus.

In contrast, *Papillibacter* and *Lachnospiraceae_ND3007_group* were negatively correlated with total protease activity, indicating that differential taxa may have diverged ecological functions in ruminal nitrogen metabolism. Particularly noteworthy, *Christensenellaceae_R-7_group* was positively correlated with total protease activity but negatively correlated with apparent NDF digestibility while also showing significant positive correlations with Lys-Thr-Lys, Ser-Leu-Ile-Gly-Lys-Val, Ginsenoside Rk3, and several ceramide-related metabolites. Previous studies have shown that enrichment of *Christensenellaceae_R-7_group* is usually associated with ruminal environments characterized by high-concentrate diets or greater nitrogen metabolic efficiency [88,89]. Combined with the present results, the association pattern of this taxon is consistent with a ruminal state characterized by differences in proteolytic activity, peptide-related metabolites, and nitrogen metabolism; however, these correlations do not establish that *Christensenellaceae_R-7_group* directly performs or regulates these processes This interpretation is consistent with its significant enrichment in the SC50 group and with the enrichment of aminoacyl-tRNA biosynthesis and protein digestion and absorption pathways in this group, suggesting that, at a moderate replacement level, the ruminal microecosystem underwent a functional adjustment characterized mainly by protein metabolic remodeling.

Among the taxa enriched in the SC75 group, *norank_f_UCG-011* was not only identified as a differential taxon in the community analysis but was also positively correlated with acetate, propionate, and apparent EE digestibility. Its metabolite association pattern was similar to that of Streptococcus, as both were positively correlated with Acetolein, Hovenolactone, and Ursolic acid. This suggests that *norank_f_UCG-011 and Streptococcus showed similar association patterns with the higher VFA concentrations and several discriminatory metabolites observed in SC75*. Combined with the metabolomic results showing strong responses in bile acid/cholesterol metabolism, membrane lipid metabolism, and triterpenoid metabolism in SC75, it may be inferred that this group established an adaptive state characterized by enhanced fermentation, active lipid and secondary metabolism, and relative metabolic network stability.

By contrast, the representative taxon *norank_f__F082* in the SC100 group was significantly negatively correlated with pH, acetate, and butyrate, and its metabolite association pattern was broadly opposite to that of *Streptococcus*. This suggests that it may represent a distinct metabolic niche under high replacement levels and may be related to changes in fermentation substrate allocation, reconstruction of material transport, and adjustment of metabolic direction, rather than simply corresponding to a VFA-producing taxon. In other words, although the SC100 group still maintained relatively high fermentation activity in some parameters, its microbiota–metabolite coupling pattern had already shown a more pronounced tendency toward restructuring and differentiation.

From the perspective of metabolite–phenotype associations, the present results also showed relatively clear functional clustering patterns. Acetolein, Hovenolactone, Emmolic acid, and Ursolic acid were significantly positively correlated with TVFA and its components, and were also positively correlated with hemicellulase activity and apparent EE digestibility, suggesting that these metabolites may be associated with stronger fermentation activity and lipid utilization status. In contrast, 3-O-Acetyloleanolic acid, Cer(18:1_3O/16:0_(2OH)), and Guavenoic acid were significantly negatively correlated with cellulase activity, apparent NDF and ADF digestibility, and VFA production, indicating that different classes of lipid- and triterpenoid-related metabolites do not carry the same functional implications for rumen status. In addition, Lys-Thr-Lys, Ser-Leu-Ile-Gly-Lys-Val, Ginsenoside Rk3, and Diosmetin-7-O-Neohesperidoside formed another representative metabolite cluster. These metabolites were positively correlated with NH_3_-N, total protease activity, apparent CP digestibility, and α-amylase activity but negatively correlated with cellulase activity and apparent ADF and NDF digestibility. These findings suggest that, under *S. cannabina* replacement, enhanced protein metabolism and enhanced fiber degradation in the rumen did not necessarily occur synchronously but instead may reflect a degree of substrate allocation bias and functional division.

The 24 h pre-slaughter feed withdrawal should also be considered when interpreting the ruminal fermentation, microbial, and metabolomic results. Prolonged feed withdrawal may alter substrate availability, fermentation activity, microbial community structure, and metabolite profiles in the rumen. Therefore, although the same fasting procedure was applied to all animals to standardize sampling conditions, the observed differences should be interpreted as treatment-associated responses under a standardized fasted state rather than as representative of the normal postprandial rumen environment.

## 5. Conclusions

Replacing alfalfa with *S. cannabina* hay did not adversely affect growth performance or metabolic health in fattening lambs while altering nutrient digestibility, ruminal fermentation, and rumen microecology. Among the replacement treatments evaluated, 75% replacement was associated with relatively coordinated changes, including improved nutrient utilization, greater bacterial diversity, enrichment of fermentation-related core taxa, and alterations in bile acid- and cholesterol-related metabolic pathways. Complete replacement induced broader microbial and metabolic changes but did not confer additional benefits in growth performance. Overall, these findings suggest that *S. cannabina* hay has potential as a partial substitute for alfalfa in diets for fattening lambs. However, because rumen samples were collected after a 24 h pre-slaughter feed withdrawal, the observed fermentation, microbial, and metabolomic differences should be interpreted as treatment-associated responses under a standardized fasted condition rather than as representative of the normal postprandial rumen environment. Given this methodological constraint, together with the relatively short experimental period and limited sample size, further studies with larger populations, longer feeding periods, and rumen sampling under normal feeding conditions or at defined time points after feeding are warranted to validate these findings and clarify the biological mechanisms and practical production value of *S. cannabina* hay.

## Figures and Tables

**Table 1:** Composition and nutrient levels of experimental diets % (DM basis).

Items	Groups				
	CON	SC25	SC50	SC75	SC100
Ingredients					
*Sesbania cannabina*	0	7.5	15	22.5	30
Alfalfa	30	22.5	15	7.5	0
Corn stalk	20	20	20	20	20
Corn	32.71	32.11	32.58	35.08	35.44
Bran	10.20	10.20	8.35	3.99	2.36
Soybean meal	3.20	3.80	4.93	6.64	7.70
Calcium Hydrogen Phosphate	0.39	0.39	0.64	0.79	1.00
Sodium Bicarbonate	0.50	0.50	0.50	0.50	0.50
NaCl	1.00	1.00	1.00	1.00	1.00
Premix ^1^	2.00	2.00	2.00	2.00	2.00
Total	100	100	100	100	100
Nutrient components					
ME ^2^	10.21	10.17	10.18	10.24	10.24
CP	12.51	12.51	12.51	12.51	12.51
MP	5.28	5.28	5.28	5.29	5.29
EE	2.63	2.66	2.65	2.59	2.59
NDF	35.70	36.02	35.95	35.40	35.37
ADF	20.04	20.38	20.59	20.60	20.82
NFC	42.29	41.68	41.56	42.04	41.86
NFC/NDF	1.18	1.16	1.16	1.19	1.18
Ca	0.87	0.81	0.83	0.82	0.83
P	0.36	0.37	0.42	0.43	0.48

CON = control group, diet containing 30% alfalfa and no *Sesbania cannabina*; SC = *Sesbania cannabina*; SC25, SC50, SC75, and SC100 = diets in which 25%, 50%, 75%, and 100% of alfalfa, respectively, were replaced with *S. cannabina* on a DM basis; ME = metabolizable energy; CP = crude protein; MP = metabolizable protein; EE = ether extract; NDF = neutral detergent fiber; ADF = acid detergent fiber; NFC = non-fiber carbohydrate; NFC/NDF = ratio of non-fiber carbohydrate to neutral detergent fiber; Ca = calcium; P = phosphorus; NaCl = sodium chloride; IU = international units. ^1^ Each kilogram of premix provides the following: vitamin A, 1400 IU; vitamin D_3_, 500 IU; vitamin E, 50 mg; Fe, 10 mg; Cu, 12.5 mg; Mn, 135 mg; Zn, 100 mg; Co, 0.5 mg; I, 1.5 mg; and Se, 0.3 mg. ^2^ ME was calculated using the equation: ME = 0.046 + 0.820 × (17.211 − 0.135 × NDF); all other nutrient values were measured.

**Table 2:** Effect of replacing alfalfa with *Sesbania cannabina* hay on growth performance of lambs.

Items	Groups						
	CON	SC25	SC50	SC75	SC100	SEM	*p*-Value ^1^
IBW, kg	22.11	22.04	22.06	22.06	22.10	0.310	1.000
FBW, kg	34.67	33.61	34.00	35.06	33.79	0.719	0.969
ADG, g/d	209.29	192.82	199.06	216.67	194.79	9.632	0.934
ADFI, kg/d	1.26 ^b^	1.19 ^b^	1.26 ^b^	1.36 ^a^	1.16 ^b^	0.017	<0.05
F/G	6.61	6.56	6.05	6.42	6.25	0.078	0.135

IBW = initial body weight; FBW = final body weight; ADG = average daily gain; ADFI = average daily feed intake; F/G = feed-to-gain ratio; SEM = standard error of the mean. Within the same row, means with different superscript letters (a, b) differ significantly (*p* < 0.05). ^1^ *p* values were calculated using one-way analysis of variance (ANOVA) (*n* = 8 per treatment group).

**Table 3:** Effect of replacing alfalfa with *Sesbania cannabina* hay on the apparent nutrient digestibility of lambs.

Items	Groups						
	CON	SC25	SC50	SC75	SC100	SEM	*p*-Value
DM, %	75.52	76.06	76.10	77.75	76.93	0.612	0.823
CP, %	61.98 ^ab^	55.95 ^b^	66.05 ^a^	68.26 ^a^	59.28 ^ab^	1.452	0.037
EE, %	48.07 ^ab^	45.62 ^b^	56.54 ^a^	58.01 ^a^	56.42 ^a^	1.590	0.028
NDF, %	52.82 ^ab^	46.85 ^b^	56.40 ^a^	53.89 ^ab^	59.19 ^a^	1.311	0.029
ADF, %	43.70 ^bc^	41.65 ^c^	50.67 ^ab^	48.08 ^abc^	53.31 ^a^	1.356	0.028

DM = dry matter; CP = crude protein; EE = ether extract; NDF = neutral detergent fiber; ADF = acid detergent fiber. Within the same row, means with different superscript lowercase letters (a, b, c) differ significantly (*p* < 0.05), whereas means sharing at least one common letter are not significantly different. *p* values were obtained using one-way analysis of variance (ANOVA), followed by Tukey’s HSD test for multiple comparisons when a significant treatment effect was detected (*n* = 8 per treatment group).

**Table 4:** Effects of replacing alfalfa with *Sesbania cannabina* hay on rumen digestive enzyme activities and rumen histomorphology in lambs.

Items	Groups						
	CON	SC25	SC50	SC75	SC100	SEM	*p*-Value
Total protease (azocasein U/(h·mL))	0.39 ^a^	0.25 ^b^	0.27 ^b^	0.35 ^a^	0.29 ^b^	0.011	<0.001
α-Amylase (μg/(min·mL))	62.35 ^b^	62.23 ^b^	71.32 ^b^	110.62 ^a^	60.66 ^b^	3.408	<0.001
Cellulase (μg/(min·mL))	22.49 ^b^	23.71 ^b^	32.98 ^a^	25.82 ^b^	32.94 ^a^	0.897	<0.001
Hemicellulase (nmol/(min·mL))	15.52 ^b^	14.36 ^b^	15.04 ^b^	23.25 ^a^	24.47 ^a^	0.813	<0.001
RPL, mm	2.11	2.07	2.20	1.73	1.87	0.128	0.794
RPW, mm	0.46	0.51	0.51	0.47	0.53	0.021	0.832
RMT, mm	1.05 ^b^	1.48 ^ab^	1.44 ^ab^	1.92 ^a^	1.69 ^a^	0.095	0.046

RPL = rumen papillae length; RPW = rumen papillae width; RMT = rumen muscular layer thickness. Within the same row, means with different superscript lowercase letters (a, b) differ significantly (*p* < 0.05), whereas means sharing at least one common letter are not significantly different. *p* values were obtained using one-way analysis of variance (ANOVA), followed by Tukey’s HSD test for multiple comparisons when a significant treatment effect was detected (*n* = 8 per treatment group).

**Table 5:** Effects of replacing alfalfa with *Sesbania cannabina* hay on rumen fermentation parameters in lambs.

Items	Groups						
	CON	SC25	SC50	SC75	SC100	SEM	*p*-Value
pH	7.30 ^a^	7.26 ^a^	7.17 ^a^	6.91 ^c^	7.05 ^b^	0.024	<0.001
TVFA (mmol/L)	62.24 ^d^	63.17 ^d^	76.53 ^c^	92.63 ^a^	85.65 ^b^	1.963	<0.001
Acetate (mmol/L)	46.01 ^d^	45.15 ^d^	54.14 ^c^	64.58 ^a^	59.50 ^b^	1.531	<0.001
Propionate (mmol/L)	11.35 ^e^	13.03 ^d^	16.14 ^c^	21.05 ^a^	19.27 ^b^	0.679	<0.001
Butyrate (mmol/L)	4.88 ^c^	4.99 ^c^	6.24 ^b^	7.00 ^a^	6.88 ^ab^	0.196	<0.001
A/P	4.07 ^a^	3.50 ^b^	3.38 ^bc^	3.10 ^c^	3.11 ^c^	0.076	<0.001
NH_3_-N (mg/dL)	25.94 ^b^	23.78 ^b^	30.22 ^a^	23.26 ^b^	17.67 ^c^	0.631	<0.001

VFA = volatile fatty acids; A:P = acetate-to-propionate ratio; NH_3_-N = ammonia nitrogen. Within the same row, means with different superscript lowercase letters (a, b, c, d, e) differ significantly (*p* < 0.05), whereas means sharing at least one common letter are not significantly different. *p* values were obtained using one-way analysis of variance (ANOVA), followed by Tukey’s HSD test for multiple comparisons when a significant treatment effect was detected (*n* = 8 per treatment group).

## Data Availability

The 16S rRNA sequencing data are available in the NCBI SRA database under BioProject accession PRJNA1458508. The untargeted metabolomics data are available in the OMIX database under accession OMIX017728.

## Supplementary Materials

The following supporting information can be downloaded at https://www.mdpi.com/article/10.3390/ani16172745/s1: Supplementary Figure S1. Effects of replacing alfalfa with different proportions of *Sesbania cannabina* on serum biochemical parameters in sheep. (A–F) Results of blood samples before feeding (Day 1); (G–L) Results after feeding (Day 60). Bars represent group means, error bars represent the standard error of the mean (SEM), and scatter points represent individual data. Different lowercase letters (a, b, c, d) above the bars indicate significant differences among different treatment groups at the same time point (*p* < 0.05), while the same letters indicate no significant difference (*p* > 0.05). Supplementary Table S1. Complete nutrient composition of two core forages (% DM basis). Supplementary Table S2. Taxonomic classification and relative abundance of rumen bacterial ASVs in lambs fed diets with different proportions of *Sesbania cannabina* hay. Columns include taxonomic ranks (Domain, Kingdom, Phylum, Class, Order, Family, Genus, Species), ASV identifier, total read count, relative percentage, and prevalence across samples. Supplementary Table S3. Effects of replacing alfalfa with different proportions of *Sesbania cannabina* hay on rumen bacterial phyla in lambs. Values are presented as means; different lowercase superscripts indicate significant differences among treatment groups (*p* < 0.05) and Supplementary Table S4. Identification information of rumen metabolites detected by untargeted metabolomics, including metabolite name, KEGG compound ID, *m*/*z*, retention time, ionization mode, adducts, molecular formula, fragmentation score, mass error, CAS ID, and relative standard deviation (RSD).
