# Supplementary material for: Effects of Replacing Alfalfa with Sesbania cannabina Hay on Growth Performance, Rumen Fermentation, and Rumen Microecology in Fattening Lambs
Source: Animals (Basel). 2026 Sep 2;16(17):2745. doi: 10.3390/ani16172745 (PMC13565084; doi:10.3390/ani16172745)
Supplement: Supplementary file 1 [file animals-16-02745-s001.zip › QC_CV_neg.pdf]

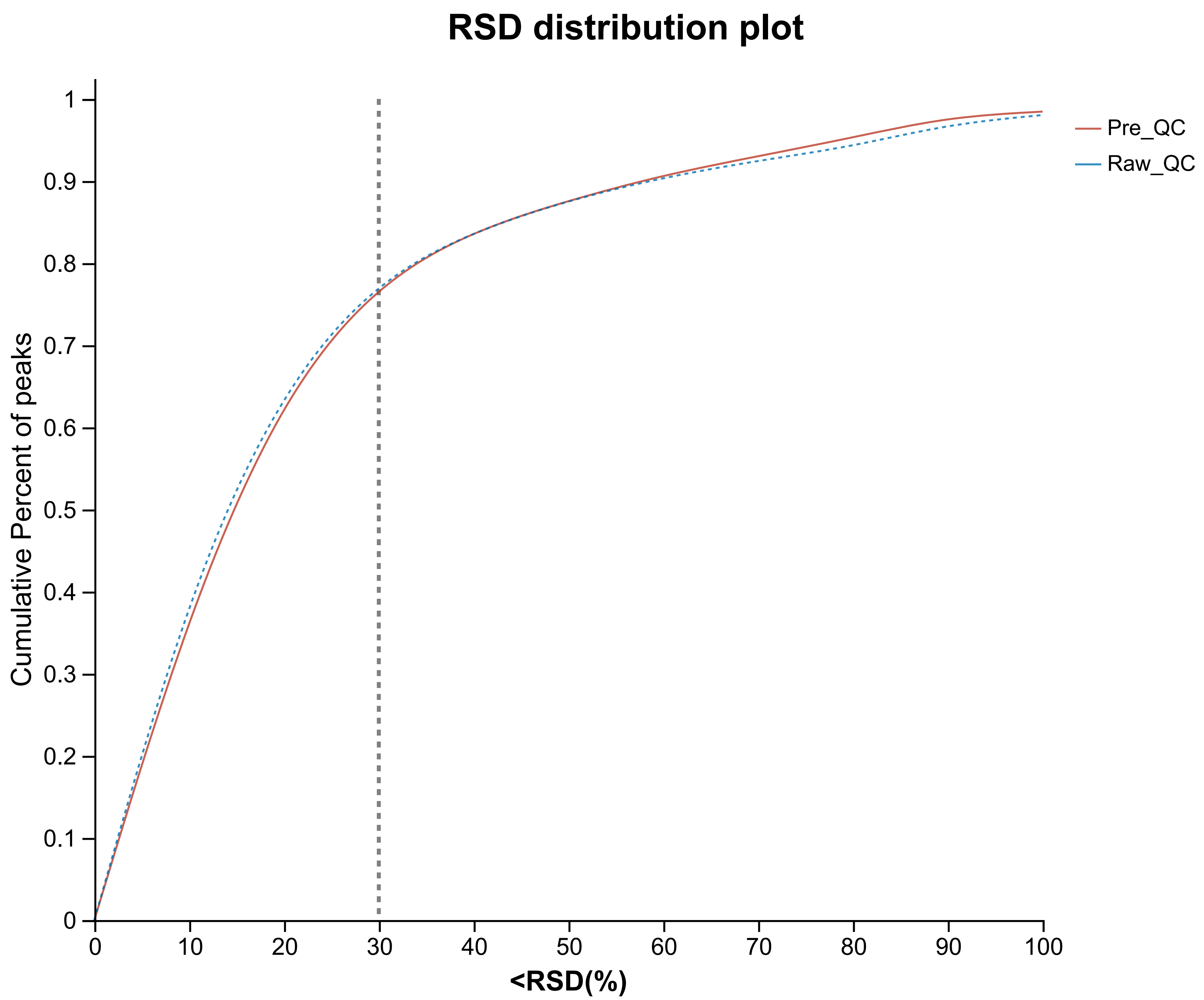
RSD distribution plot
Cumulative Percent of peaks
<RSD(%)
Pre_QC
Raw_QC
0
0.1
0.2
0.3
0.4
0.5
0.6
0.7
0.8
0.9
1
0
10
20
30
40
50
60
70
80
90
100
