# Supplementary material for: Effects of Replacing Alfalfa with Sesbania cannabina Hay on Growth Performance, Rumen Fermentation, and Rumen Microecology in Fattening Lambs
Source: Animals (Basel). 2026 Sep 2;16(17):2745. doi: 10.3390/ani16172745 (PMC13565084; doi:10.3390/ani16172745)
Supplement: Supplementary file 1 [file animals-16-02745-s001.zip › Rarefaction curve (Sobs index at genus level).pdf]

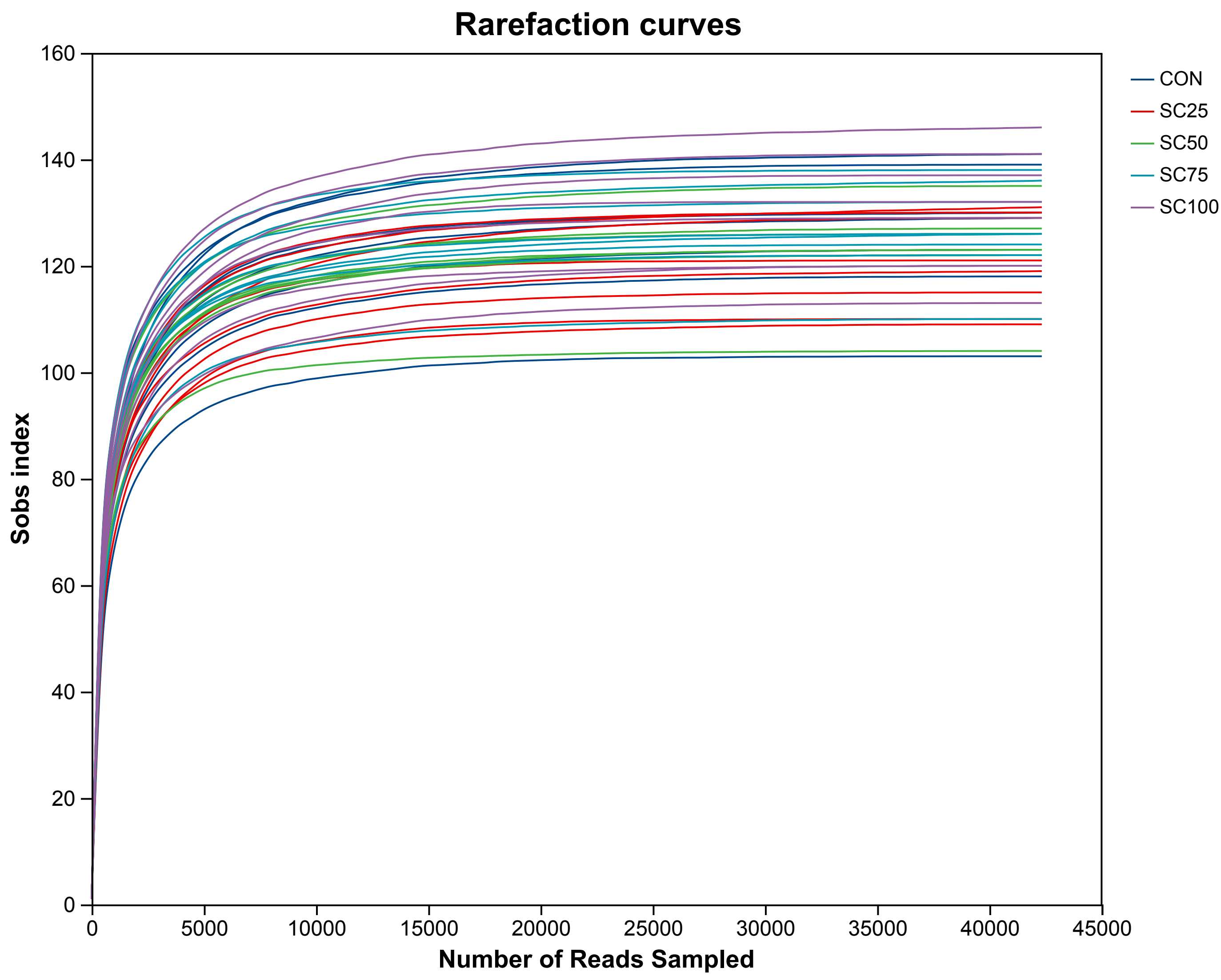
Rarefaction curves
Sobs index
0
20
40
60
80
100
120
140
160
0
5000
10000
15000
20000
25000
30000
35000
40000
45000
Number of Reads Sampled
CON
SC25
SC50
SC75
SC100
