# Supplementary material for: Effects of Replacing Alfalfa with Sesbania cannabina Hay on Growth Performance, Rumen Fermentation, and Rumen Microecology in Fattening Lambs
Source: Animals (Basel). 2026 Sep 2;16(17):2745. doi: 10.3390/ani16172745 (PMC13565084; doi:10.3390/ani16172745)
Supplement: Supplementary file 1 [file animals-16-02745-s001.zip › Supplementary_Figure_S1.pdf]

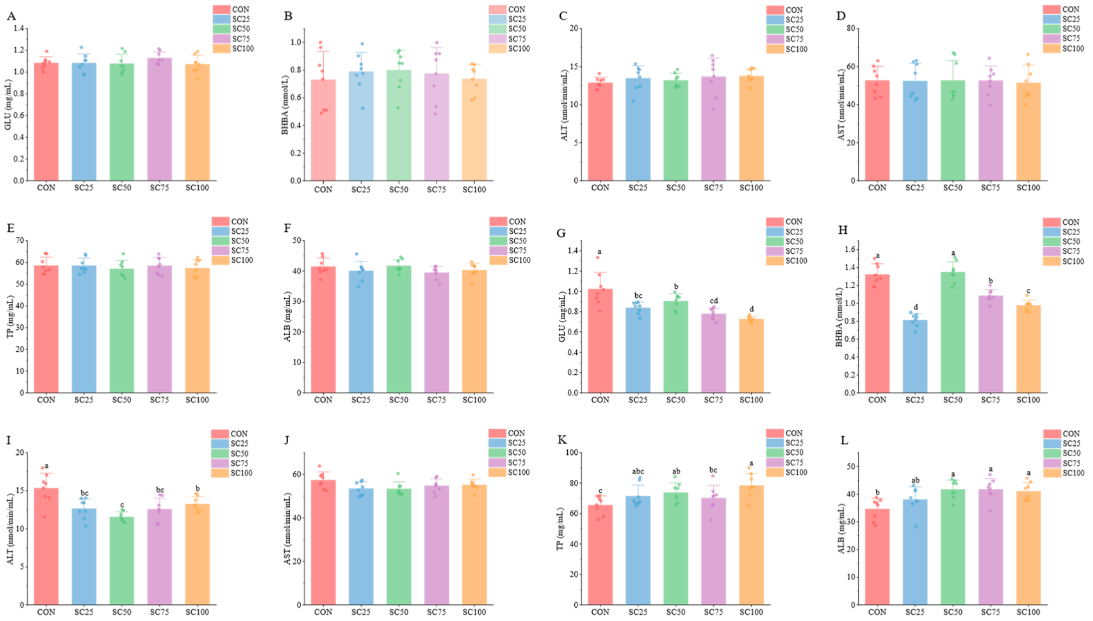

Figure S1. Effects of replacing alfalfa with different proportions of *Sesbania cannabina* on serum biochemical parameters in sheep. (A-F) Results of blood samples before feeding (Day 1); (G-L) Results after feeding (Day 60). Bars represent group means, error bars represent the standard error of the mean (SEM), and scatter points represent individual data. Different lowercase letters (a, b, c, d) above the bars indicate significant differences among different treatment groups at the same time point ($P < 0.05$), while the same letters indicate no significant difference ($P > 0.05$).
