# Supplementary material for: Effects of Replacing Alfalfa with Sesbania cannabina Hay on Growth Performance, Rumen Fermentation, and Rumen Microecology in Fattening Lambs
Source: Animals (Basel). 2026 Sep 2;16(17):2745. doi: 10.3390/ani16172745 (PMC13565084; doi:10.3390/ani16172745)
Supplement: Supplementary file 1 [file animals-16-02745-s001.zip › Supplementary_Table_S1.pdf]

Table S1. Complete Nutrient Composition of Two Core Forages % (DM basis).

Nutrient components	Alfalfa hay	*Sesbania cannabina* hay
DM	93.6	93.5
CP	18.0	15.0
EE	1.70	2.2
NDF	45.7	49.8
ADF	29.7	33.9
Ash	13.0	6.26
Ca	1.86	1.11
P	0.18	0.29
MP	8.1	6.6

DM = dry matter; CP = crude protein; EE = ether extract; NDF = neutral detergent fiber; ADF = acid detergent fiber; Ca = calcium; P = phosphorus; MP = metabolizable protein.
