# Supplementary material for: Effects of Replacing Alfalfa with Sesbania cannabina Hay on Growth Performance, Rumen Fermentation, and Rumen Microecology in Fattening Lambs
Source: Animals (Basel). 2026 Sep 2;16(17):2745. doi: 10.3390/ani16172745 (PMC13565084; doi:10.3390/ani16172745)
Supplement: Supplementary file 1 [file animals-16-02745-s001.zip › Supplementary_Table_S3.pdf]

Table S3. Effects of replacing alfalfa with different proportions of *Sesbania cannabina* on rumen bacterial phyla in lambs

Items	Groups						
	CON	SC25	SC50	SC75	SC100	SEM	*P*-value
Bacteroidota (%)	48.86[ab]	52.02[a]	49.90[ab]	44.04[c]	43.88[c]	1.046	0.036
Bacillota (%)	44.01	41.86	43.71	49.21	49.36	1.146	0.119
Spirochaetota (%)	7.06	3.98	4.15	6.41	6.61	0.570	0.269
